# Simultaneous de novo calling and phasing of genetic variants at chromosome-scale using NanoStrand-seq

**DOI:** 10.1038/s41421-024-00694-9

**Published:** 2024-07-09

**Authors:** Xiuzhen Bai, Zonggui Chen, Kexuan Chen, Zixin Wu, Rui Wang, Jun’e Liu, Liang Chang, Lu Wen, Fuchou Tang

**Affiliations:** 1 Biomedical Pioneering Innovation Center (BIOPIC), Peking University, Beijing, China; 2 Beijing Advanced Innovation Center for Genomics (ICG), Ministry of Education Key Laboratory of Cell Proliferation and Differentiation, Beijing, China; 3 Changping Laboratory, Beijing, China; 4 Peking-Tsinghua Center for Life Sciences, Academy for Advanced Interdisciplinary Studies, Peking University, Beijing, China; 5 School of Life Sciences, Peking University, Beijing, China; 6 Department of Medicine, Cancer Institute, Stanford University, Stanford, CA USA; 7 State Key Laboratory of Female Fertility Promotion, Center for Reproductive Medicine, Department of Obstetrics and Gynecology, Peking University Third Hospital, Beijing, China; 8 National Clinical Research Center for Obstetrics and Gynecology (Peking University Third Hospital), Beijing, China; 9 Key Laboratory of Assisted Reproduction (Peking University), Ministry of Education Beijing, Beijing, China; 10 Key Laboratory of Reproductive Endocrinology and Assisted Reproductive Technology, Beijing, China

**Keywords:** Structural variation, Bioinformatics

## Abstract

The successful accomplishment of the first telomere-to-telomere human genome assembly, T2T-CHM13, marked a milestone in achieving completeness of the human reference genome. The upcoming era of genome study will focus on fully phased diploid genome assembly, with an emphasis on genetic differences between individual haplotypes. Most existing sequencing approaches only achieved localized haplotype phasing and relied on additional pedigree information for further whole-chromosome scale phasing. The short-read-based Strand-seq method is able to directly phase single nucleotide polymorphisms (SNPs) at whole-chromosome scale but falls short when it comes to phasing structural variations (SVs). To shed light on this issue, we developed a Nanopore sequencing platform-based Strand-seq approach, which we named NanoStrand-seq. This method allowed for de novo SNP calling with high precision (99.52%) and acheived a superior phasing accuracy (0.02% Hamming error rate) at whole-chromosome scale, a level of performance comparable to Strand-seq for haplotype phasing of the GM12878 genome. Importantly, we demonstrated that NanoStrand-seq can efficiently resolve the MHC locus, a highly polymorphic genomic region. Moreover, NanoStrand-seq enabled independent direct calling and phasing of deletions and insertions at whole-chromosome level; when applied to long genomic regions of SNP homozygosity, it outperformed the strategy that combined Strand-seq with bulk long-read sequencing. Finally, we showed that, like Strand-seq, NanoStrand-seq was also applicable to primary cultured cells. Together, here we provided a novel methodology that enabled interrogation of a full spectrum of haplotype-resolved SNPs and SVs at whole-chromosome scale, with broad applications for species with diploid or even potentially polypoid genomes.

## Introduction

A haplotype refers to a grouping of genetic variants that occur along a single chromosome and tend to be inherited together. Haplotype phasing information is pivotal for a comprehensive understanding of genetic diversity and its connection to disease. Genetic variants can be physically linked to each other on the same chromosome, accounting for various scenarios such as genetic inheritance patterns, allele-specific gene expression^1^, drug sensitivity^2^, and tumor susceptibility^3^. Delineating the full landscape of haplotype information would profoundly deepen our understanding of the relationships between genomic differences and physiological/pathological phenotypes in an organism^1^. Indeed, haplotype phasing has long been a hot topic in genome research field^1,4^. Numerous available techniques mainly focused on phasing of single nucleotide polymorphisms (SNPs). Strand-seq, a short-read sequencing technology^5^, is the gold standard for global phasing and has succeeded in directly phasing SNPs at the whole-chromosome scale without the need for pedigree data^6^. It was widely acknowledged that structural variations (SVs, ≥ 50 bp) play critical roles for gene expression regulation and maintenance of genome stability. However, systematically detecting and phasing deletions and insertions remained challenging for Strand-seq technique due to its innate limitation of short length of reads. Furthermore, over 15% of genomic regions characterized by atypical GC contents or long runs of repetitive elements are highly enriched for SVs, which pose a severe challenge to short-read mapping technologies^7–9^. In addition, SVs are prevalent not only among healthy human populations^8^, but also responsible for numerous diseases, such as cancer^10^, congenital abnormalities^11^, and cognitive disabilities^12^. SVs affect genomic architectures or *cis*-regulatory elements across a larger span of nucleotides compared to SNPs. Therefore, elucidating all combinations of genetic variations within the regulatory elements and the coding regions (i.e., discriminating between *cis*- and *trans*-relationships between genetic variants) of a given genome is essential for allele-specific expression analysis and other diplopic effect-related analysis^13–15^, which is important for genomic diagnosis^1,16^.

Excitingly, burgeoning single-molecule sequencing (SMS, also known as third-generation sequencing, TGS) platforms, such as Pacific Biosciences (PacBio) and Oxford Nanopore Technologies (ONT), can generate read lengths increased by 1–2 orders of magnitude compared to short-read next-generation sequencing (NGS) platforms. Emerging experimental and computational methodologies based on SMS platforms substantially advanced our capacity to detect SVs, especially those in complex genomic regions, largely attributed to improvement in genome assembly and haplotype phasing. However, despite these advances, existing approaches have intrinsic drawbacks. For instance, some computational approaches heavily relied on pedigree information^17^, yet it remained challenging to phase heterozygous SNPs (hetSNPs) of offspring when both parents were heterozygous of corresponding SNP loci. In addition, quite often parental materials were not available, making these strategies practically infeasible. Other methods for haplotype phasing resulted in long-range haplotype blocks, but still missed phase information across the genomic regions of very long homozygous sequences or typically required integrating scaffolding data (link-reads^18,19^ or Hi-C^20,21^ reads) to provide long-range genetic linkage information^22^. For example, the combination of short-read-based Strand-seq and PacBio long-read bulk sequencing data improved the ability of haplotype phasing of both SNPs and SVs^23^. However, all of the tough genomic regions, such as those with dense hetSNPs, long genomic regions of homozygosity (without any hetSNP in a long genomic region), centromeres, pericentromeric regions, and other repetitive sequences^9,24,25^, posed severe obstacles for haplotype phasing at the whole-chromosome scale.

To address these issues, we developed an approach, called NanoStrand-seq, by systematically modifying the short-read-based Strand-seq method^5,6,26,27^ to make it compatible with the SMS platforms. NanoStrand-seq was a technique that leveraged a single adaptor-embedded Tn5 enzyme, specifically enriching long genomic DNA fragments in an individual cell. The template strands were specifically labeled by two rounds of primer extension reactions. We also developed a robust pipeline tailored for NanoStrand-seq data. Our results demonstrated that NanoStrand-seq was capable of direct de novo calling and phasing of SNPs with excellent performance, which was comparable to Strand-seq. Moreover, SNPs in hyper-polymorphism major histocompatibility complex (MHC) region could be readily detected and phased using NanoStrand-seq. By combining with known SNPs that were directly linked to NanoStrand-seq-derived hetSNPs through long fragments, as few as 100 NanoStrand-seq libraries achieved a high calling sensitivity and phasing accuracy (95.35% of recall rate and 0.29% of Hamming error rate). More importantly, NanoStrand-seq could pinpoint haplotype-specific complex SVs. NanoStrand-seq was proved to be a powerful method to obtain highly accurate haplotype-resolved genome assembly when integrating with bulk long-read-based sequencing approaches.

## Results

### Development and characterization of NanoStrand-seq

**Fig. 1:**
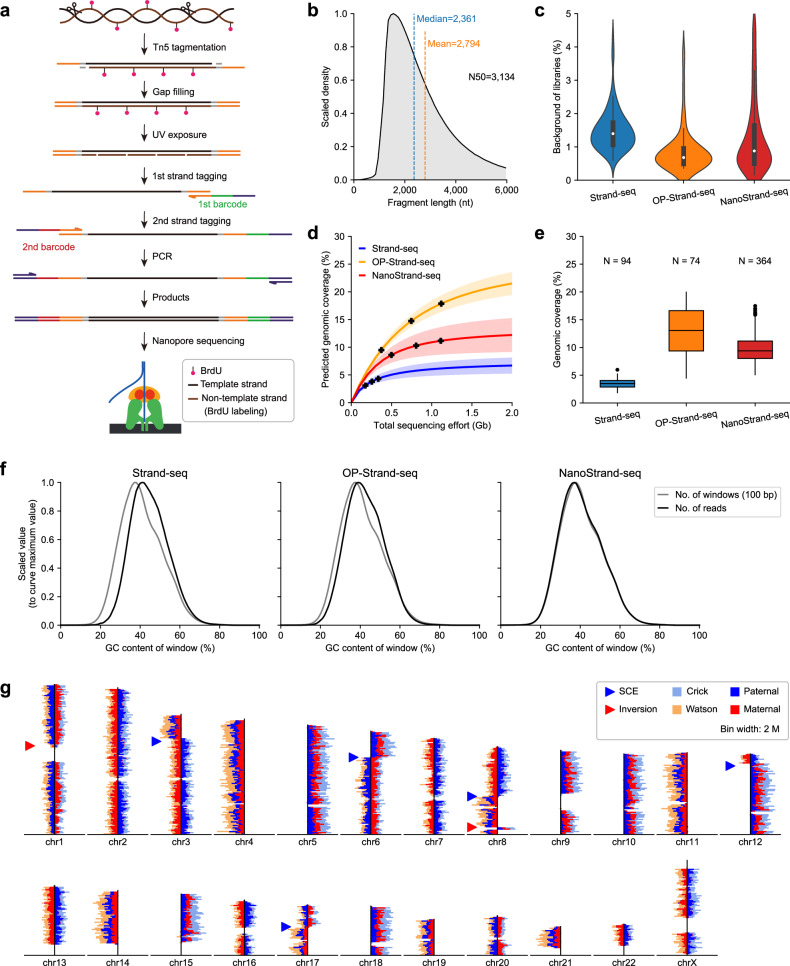
Strategy and characterization of NanoStrand-seq. **a** Schematic procedure of NanoStrand-seq. **b** Length distribution of inserted fragment of NanoStrand-seq. **c** Background of libraries produced by Strand-seq, OP-strand-seq, and NanoStrand-seq. **d** The fitted complexity (genomic coverage) curves for libraries produced by Strand-seq, OP-Strand-seq, and NanoStrand-seq. Solid lines and shaded areas indicate mean and SD, respectively. The corresponding sequencing efforts of libraries were shown as black spots (the middle black spot indicates the mean of the sequencing efforts, and the corresponding left and right black spots indicate SD). **e** Comparison of genomic coverage of Strand-seq, OP-Strand-seq, and NanoStrand-seq. **f** Plots of GC bias for different methods. The gray lines indicate the relative numbers of different GC content windows in the genome, and the black lines show the relative quantities of reads with different GC contents detected by each method. **g** Representative ideogram plot of a NanoStrand-seq library distinguishing three possible template strand inheritance patterns (WW, CC, CW) and visible SCEs and inversions. Directional read counts were plotted as horizontal bars for each chromosome. The parental origin of reads was annotated by GIAB hetSNPs.

We aimed to develop NanoStrand-seq for capturing template strand-specific long genomic DNA fragments compatible with SMS platforms. Similar to short-read-based Strand-seq, the key step of cell preparation was that 5′-bromo-2′-deoxyuridine (BrdU; a thymidine analog)-substituted newly synthesized DNA strands in daughter cells were targeted to be excised and removed, ensuring only template DNA strands being amplified (Fig. 1a; Supplementary Fig. S1a). Major modifications compared to the Strand-seq are as follows. Firstly, for Strand-seq, genomic DNA was cleaved by MNase, which was suitable for obtaining short DNA fragments (100–300 bp). Instead, NanoStrand-seq initiated with low-density Tn5 transposon embedded with one adaptor, instead of a pair of adaptors, for effective fragmentation of the single-cell genomic DNA, showing a more concentrated distribution of long genomic DNA fragments and superior robustness across different single-cell libraries (Fig. 1b; Supplementary Fig. S1b, c). Notably, Tn5 transposase was hyperactive and stable, ensuring highly robust fragmentation of genomic DNAs. Secondly, during initial testing process, we found that the efficiency of ligation strategy using Y-shaped adaptors, which was used in the short-read-based Strand-seq method, decreased drastically in long DNA fragment ligation. This strategy yielded a very low amount of DNA products with predominant shorter fragments and primer dimers. We speculated that this phenomenon may be due to the decreased number of genomic DNA fragments (as DNA fragments are generally longer) from a single cell. Herein, after the dissociation of Tn5 from the tagmented DNA ends, we performed a gap-filling reaction, followed by two rounds of primer extension reactions to achieve efficient strand-specific tagging. The extension primers incorporated a fixed sequence and a 24-nt barcode compatible with the ONT sequencing platform. Distinct combinatorial barcodes on the 5’ and 3’ ends of genomic DNAs preserved strand-specific information (Supplementary Fig. S1d and Table S1). Thirdly, genomic DNA fragments tagged by unique combinatorial barcodes were subjected to optimized PCR amplification and subsequently pooled together to microgram amount for Nanopore sequencing. In particular, by tagging similar sequences at both ends of genomic DNA fragments through one adaptor-embedded Tn5 tagmentation, an intramolecular hairpin structure is more likely to form within short DNA fragments, reducing their amplification and effectively enriching longer genomic DNA fragments (Supplementary Fig. S1c)^28–30^.

We first applied NanoStrand-seq to the well-characterized human diploid cell line GM12878, which was a female human B lymphoblastoid-derived cell line. Given the inherent less-accuracy of ONT sequencing reads, we tested and determined that DNA barcodes with no more than an edit distance of 5 were reliable to assign specific combinatorial barcodes for each cell (Supplementary Fig. S2a–f). After trimming exogenous sequences, removing chimeric reads, and filtering out low-quality alignments, we showed that 64.24% of the total reads were successfully retrieved for further analyses (Supplementary Fig. S2e–h), yielding genomic DNA fragments of average length 2794 bp (N50 of 3134 bp) (Fig. 1b). The length of DNA fragments were ~20 times longer than reads derived from the short-read-based Strand-seq. The average length of the top 10% mapped reads was 6335 bp. To initially assess the quality of NanoStrand-seq libraries, we performed side-by-side comparisons with Strand-seq and OP-Strand-seq (a high-throughput version of Strand-seq)^31^. During library construction, both insufficient infiltration of BrdU and incomplete degradation of newly formed DNA strands may result in background noises, which can affect the accuracy of haplotype-specific reads assigned and consequently lead to phasing errors. Here, we observed that NanoStrand-seq displayed a median background of 0.88% per cell, comparable to 0.68% of OP-Strand-seq, lower than 1.40% of Strand-seq (Fig. 1c; Supplementary Fig. S3), implying that NanoStrand-seq libraries were of high quality. Moreover, NanoStrand-seq exhibited 9.39% (median) of genomic coverage per cell, higher than that of Strand-seq (3.51%) and comparable to that of OP-Strand-seq (13.05%) (Fig. 1d, e). Notably, NanoStrand-seq displayed superior even-coverage across the whole genome, the mean GC content of NanoStrand-seq data was 40.75%, very close to 40.9% reported for the human reference genome^32^, with barely GC bias in comparison with Strand-seq (Fig. 1f; Supplementary Fig. S4a, b). As an illustration, we observed the template strand inherited patterns for each chromosome in a single-cell NanoStrand-seq library, in which a few randomly distributed sister chromatid exchanges (SCEs) and known inversion events were also observed. These results collectively confirmed the success of our experimental strategies (Fig. 1g). Finally, we obtained 364 single-cell NanoStrand-seq libraries (44.07%, 364/826) after filtering with stringent criteria (with ≥ 80,000 unique reads and < 5% background; shown in Supplementary Fig. S3b). The average sequencing depth of these cells that passed quality control was 0.27×, with a mean duplicate rate of 60.37% (Supplementary Fig. S2i). We randomly selected 350 of them for the downstream analyses.

### Detection and characterization of genomic inversions by NanoStrand-seq

**Fig. 2:**
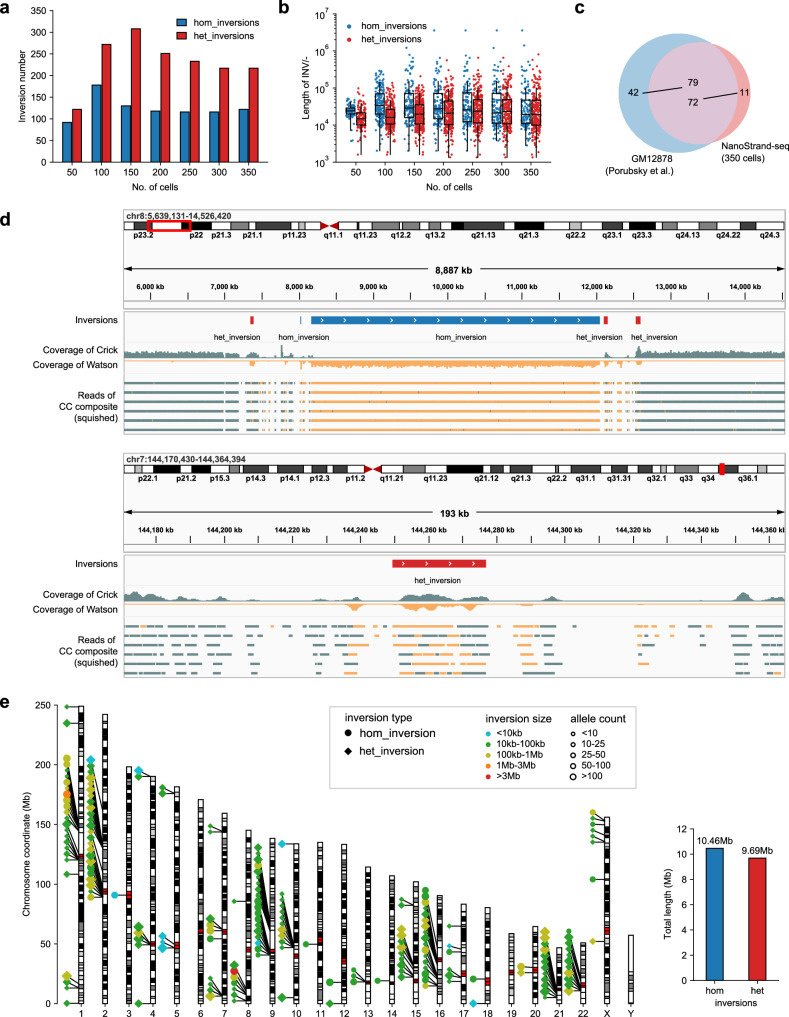
Inversions detected by NanoStrand-seq. **a** Histogram displaying the number of putative homozygous inversions and putative heterozygous inversions detected by NanoStrand-seq at a given cell number. **b** Box plot showing the length of putative homozygous inversions and putative heterozygous inversions detected by NanoStrand-seq at the given cell number. Each spot denotes a single inversion event. **c** Venn diagram showing the consistency between the confident inversions detected by NanoStrand-seq and inversion call set of GM12878 of Porubsky et al. 2022 in *Cell*^34^ (presumed to be the ground truth for GM12878 inversions). **d** Examples of the putative homozygous inversion located on Chr8 (top) and putative heterozygous inversion located on Chr7 (bottom) detected by NanoStrand-seq. **e** Ideogram plot displaying putative inversions on each chromosome^34^ detected by NanoStrand-seq (left panel). The total length of putative homozygous inversions and putative heterozygous inversions were shown in the right panel.

Prior to achieving highly accurate haplotype phasing, it is essential to address and eliminate certain confounding factors. For instance, genomic inversions represent a frequent source of errors in haplotype phasing. Similar to short-read-based Strand-seq (a prominent method for inversion calling), inversions in NanoStrand-seq were also visualized as strand orientation changes relative to the neighboring reads in the chromosome ideogram (Fig. 2d; Supplementary Fig. S5a)^33^. We optimized the bioinformatic pipeline developed for Strand-seq^33^ to systematically de novo detect genome-wide inversion events (termed putative inversions in this study) relative to GRCh38 (Supplementary Fig. S5b–e). We observed, for instance, a 3.89-Mb recurrent homozygous inversion on Chr8, consistent with previous reports^34,35^, a 27.43-kb heterozygous inversion on Chr7 (Fig. 2d), and two complex translocation events supported by linked fragments (Supplementary Fig. S6). Overall, we identified 339 putative inversions using 350 individual cells. These putative inversions were enriched at peri-centromere regions and abundant throughout the whole genome (Fig. 2a, b, e). It was noted that inversion calling remained challenging as to genomic regions of high complexity. To date, there was no comprehensive curated set of inversion call sets and no available practice for automatically filtering false positive inversions and performing benchmark analysis. Upon our manual inspection, 83 high confident inversions remained. 86.8% (72/83) of them were consistent with those obtained by previous study (Fig. 2c)^34^. Detection of genomic inversions was a critical step to prepare for the following whole-chromosome haplotype phasing. Therefore we recommended carefully chosen criteria to augment the sensitivity of inversion detection, which probably led to an overestimation of inversion events. In the intermediate step of haplotype phasing mentioned below, the phase information of genetic variants located within putative homozygous inversion regions would be reversed, while genetic variants within the putative heterozygous inversion regions would be excluded.

### Reconstruction of whole-chromosome scale haplotypes by NanoStrand-seq

**Fig. 3:**
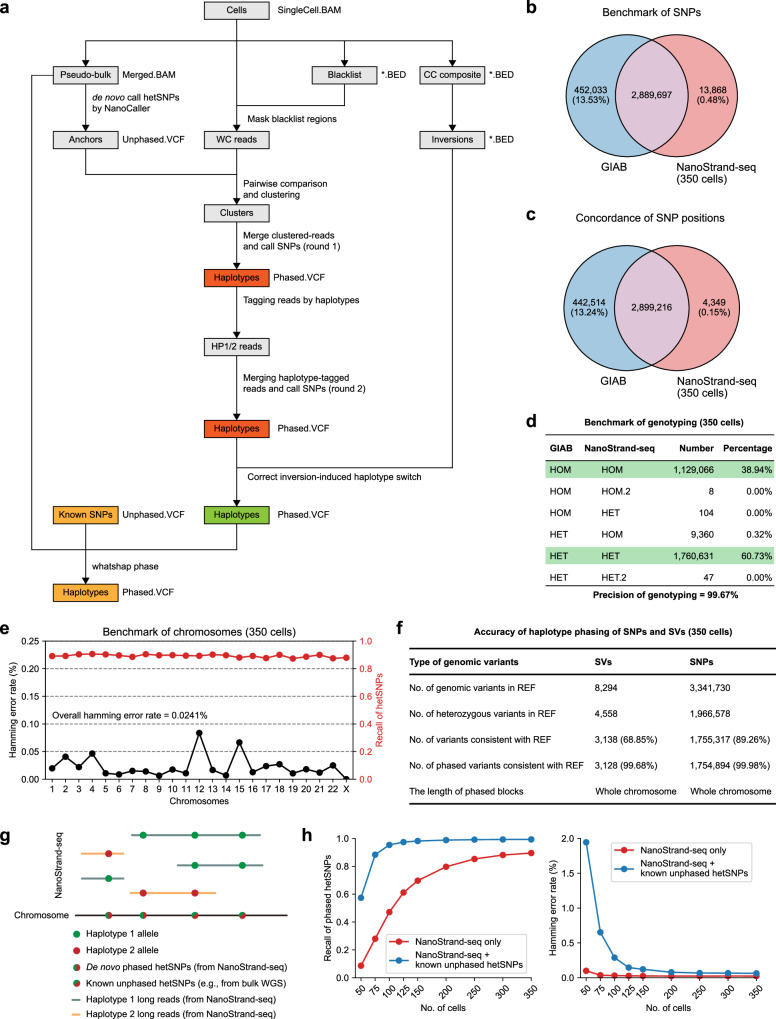
The strategy of de novo calling and phasing of hetSNPs across the whole genome. **a** Pipeline for two-round haplotype reconstructions to call more accurate and complete haplotype-resolved SNPs. Calling referred to the process of identifying SNPs relative to the reference genome (GRCh38). **b** The consistency of SNP calling between NanoStrand-seq and GIAB. **c** The consistency of SNP position between NanoStrand-seq and GIAB. **d** The precision of genotyping using NanoStrand-seq. Genotyping referred to the process of determining the genotype at each locus for the homologous allele. **e** The Hamming error rate and recall rate of hetSNPs on each chromosome. We used the GIAB dataset as a benchmark. **f** Total number of detected and phased SVs (including both insertions and deletions) and SNPs compared to the reference genome (GRCh38). The genotyping and phasing of reference SVs were determined by PacBio CCS reads and the nearest hetSNPs annotated by GIAB. **g** Schematic of phasing with known hetSNPs (derived from GIAB) using NanoStrand-seq long reads. **h** Recall rate and Hamming error rate of de novo phasing hetSNPs by NanoStrand-seq only and a strategy that NanoStrand-seq combined with known hetSNPs (from GIAB) that linked in the same reads with hetSNPs called by NanoStrand-seq.

High precision of de novo SNP calling was a prerequisite for effective haplotype phasing. Our analysis revealed that 53.79% of covered genomic regions were covered at least twice in each haplotype per cell (Supplementary Fig. S7a). Further investigation indicated that high precision of phasing was feasible when there were at least two supporting reads at the same loci for each haplotype (Supplementary Fig. S7b, c). Subsequently, we designed a pipeline to independently reconstruct the haplotype-resolved genomic sequences (Fig. 3a). In detail, we first called hetSNPs from 350 merged NanoStrand-seq libraries (pseudo-bulk). These hetSNPs served as anchors to cluster the template strand of individual cells with WC patterns (both Watson and Crick inherited template strands) for each chromosome (Supplementary Fig. S8). Accordingly, haplotype-specific BAM files were generated. Then we performed de novo SNP calling for each haplotype using haplotype-specific BAM files under stringent filter criteria, thus generating Haplotype 1 (HP1) and Haplotype 2 (HP2) sequences (Supplementary Fig. S9). This process was defined as Round 1 haplotype reconstruction. Subsequently, using these phased hetSNPs from Round 1 reconstruction, we re-annotated the original reads of each NanoStrand-seq library and extracted genomic regions in which reads were exclusively assigned to one of the consensus haplotypes. Subsequently, we repeated the SNP calling process. Note that analytical confounders including putative genomic inversions, SCE events, and aneuploidy (gain or loss) were successfully either precluded or amended (Supplementary Fig. S10a). This process was defined as Round 2 haplotype reconstruction.

Ultimately, a total of 3,241,663 SNPs were de novo called throughout the entire genome using 350 NanoStrand-seq libraries, with each SNP supported by two homologs simultaneously (Supplementary Fig. S10e). To evaluate the accuracy and completeness of SNPs defined in our study, we benchmarked SNPs (a total of 2,903,565) within high-confidence genomic regions annotated by GIAB^36,37^. We found that de novo SNP calling exhibited a high precision (99.52%) and recall rate (86.47%) relative to GIAB, respectively (Fig. 3b). Remarkably, NanoStrand-seq displayed superior genotyping precision (99.67%) (Fig. 3c, d), along with high-quality phasing performance (with 0.02% of overall Hamming error rate) (Fig. 3e, f; Supplementary Table S3). It was noteworthy that Round 2 reconstruction not only increased the recall rate of SNP calling but also improved phasing accuracy as the cell number increased compared to Round 1 reconstruction, up to 350 cells (Supplementary Fig. S10b, c). Clearly, SNPs called by NanoStrand-seq were well distributed across the human genome, with an extremely high SNP density in the well-known MHC region as expected (Supplementary Fig. S10d).

In addition, considering the high sequencing cost of the Nanopore platform, we down-sampled the number of cells sequenced to determine the minimum cell number required for accurate phasing of hetSNPs. Without a doubt, we found that the recall rate of hetSNPs gradually increased when the number of sequenced cells increased, and the overall Hamming error rate decreased gradually (Fig. 3g, h; Supplementary Table S2). Supplementing with known SNPs (in this study, we used hetSNPs derived from the GIAB truth set and removed the phase information; otherwise, we recommended high-quality short-read sequencing data for accurate variant calling if the gold standard was unavailable) that linked in the same reads with hetSNPs called by NanoStrand-seq (Fig. 3g), 350 individual cells exhibited high hetSNP recall rate (99.33%) and phasing performance (0.06% of Hamming error rate), while as few as 100 individual cells exhibited comparable performance (95.35% of recall rate, and 0.29% of Hamming error rate) (Fig. 3h). From this perspective, we concluded that NanoStrand-seq is a powerful tool for genome-wide SNP calling, genotyping, and haplotype phasing at the whole-chromosome scale.

### Extensive evaluations of NanoStrand-seq performance in high-complexity genomic regions

**Fig. 4:**
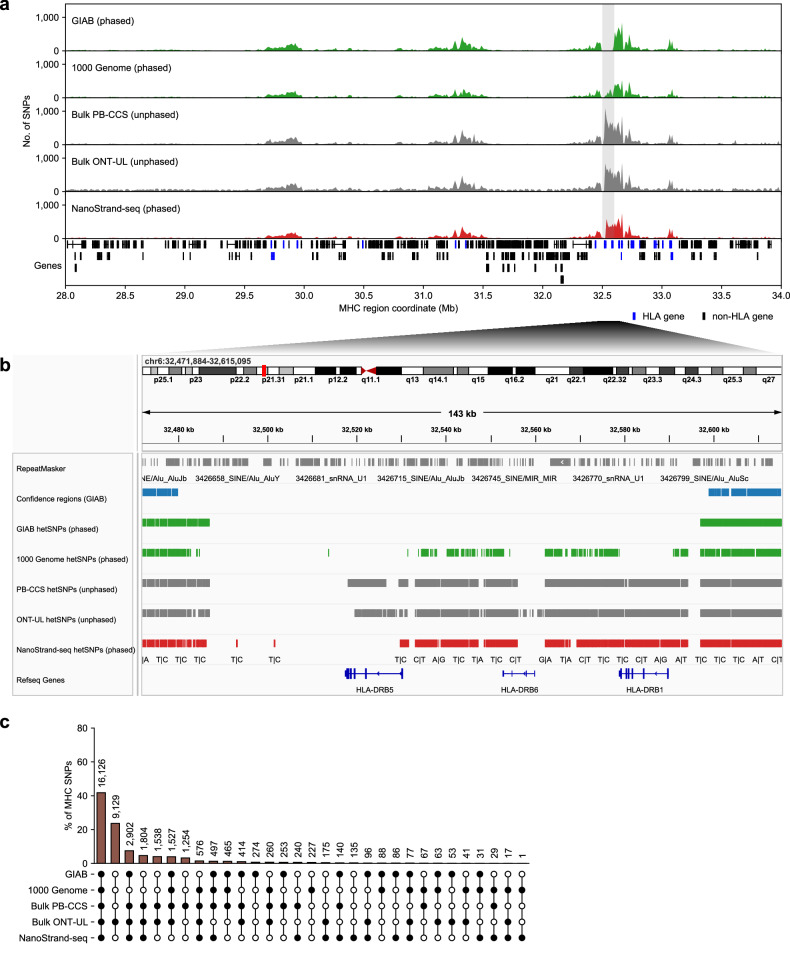
The performance of NanoStrand-seq in calling SNPs within high-complexity genomic regions. **a** SNP distribution within five different SNP call sets (top) and gene structure (bottom) in the MHC region. The light gray column highlights additional SNPs detected by NanoStrand-seq relative to GIAB, while most of these SNPs were overlapped with 1000 Genome, PB-CCS data, or bulk ONT-UL data. **b** The concordance of SNPs in the highlighted area (**a**) in five different SNP call sets. **c** The concordance of SNPs in the MHC region in the five different SNPs call sets.

To assess the effectiveness and robustness of the NanoStrand-seq approach, we evaluated its performance in haplotype phasing within highly complex genomic regions rich with informative SNPs. We first dissected the MHC region^38^, a genomic region spanning ~6 Mb on the short arm of chromosome 6. MHC region was medically important in almost all known autoimmune diseases and organ transplantations^39,40^. It was highly divergent between individuals and showed significant differences from the reference genome, which made it exceptionally difficult to be accurately mapped by short-read sequencing^41^. As such, genomic phasing of the entire MHC region was critical yet very challenging. The previous reports addressed this issue by locus-specific amplification and integration of multi-datasets including linked reads, ONT and PB-CCS reads. Yet even so, MHC phasing was still not complete^41^. It was important to note that we benchmarked the whole region of MHC, instead of only the GIAB-defined high-confidence regions. We showed that NanoStrand-seq retrieved 86.42% of SNPs (87.02% of precision rate), accurately genotyped (99.55% of precision rate) and phased MHC locus (0% Hamming error rate), compared to GIAB, respectively (Supplementary Fig. S11a–c). Extensive analysis documented that the SNP profile of NanoStrand-seq was highly consistent with the integrated datasets including 1000 Genome^42^, PB-CCS, and ONT-UL (i.e., Nanopore ultra-long sequencing data) (Fig. 4a–c; Supplementary Fig. S11a–e). Specifically, we found high calling consistency among HLA genes which enriched with a large number of repetitive elements, compared with hetSNPs from other datasets (Supplementary Figs. S11f, S12). In addition, we further showed that NanoStrand-seq could efficiently reconstruct SNPs with phase information among other gene-rich regions such as the olfactory receptor (*OR*) repertoire, the entire *TCR* repertoire, and the *BCR* repertoire, *IGK* and *IGH* repertoire (Supplementary Fig. S13 and Table S4). Taken together, NanoStrand-seq was a powerful approach for building accurate haplotypes for hetSNPs at whole-chromosome scale, which was particularly beneficial for highly polymorphic genomic regions.

### Direct calling and phasing of SVs across the whole genome by NanoStrand-seq

**Fig. 5:**
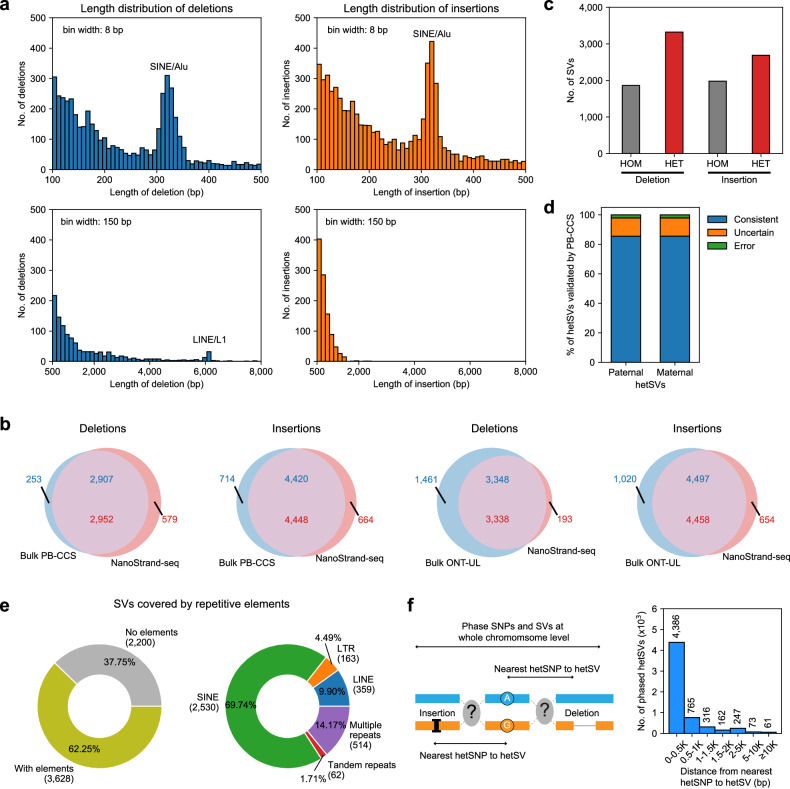
The performance of NanoStrand-seq in detecting and phasing SVs. **a** Length distribution of deletion (left) and insertion (right) events. **b** Venn diagram illustrating the consistency of SVs between NanoStrand-seq, bulk PB-CCS, and bulk ONT-UL. **c** The number of SVs derived from NanoStrand-seq. **d** The consistency of phased hetSVs between NanoStrand-seq and PB-CCS data. The parental information was annotated by GIAB. **e** The pie charts showing the proportion of SVs that contained repetitive elements (left) and the type of repetitive elements (right). **f** The distance distribution of haplotype-resolved SVs to its nearest hetSNPs. The schematic diagram on the left depicts a hypothetical situation.

Beyond SNP phasing, we explored and articulated the unique advantage of NanoStrand-seq for direct genome-wide SV calling and phasing compared to short-read-based Strand-seq. First, we proceeded to de novo call SVs using merged NanoStrand-seq data (pseudo-bulk from 350 single-cell libraries) and assessed the fidelity of the SV call set. A total of 21,211 deletion and insertion events were identified (Supplementary Fig. S14c). Substantial repetitive elements were included in the NanoStrand-seq SV call set, encompassing clear peaks at 300 bp and 6 kb, probably representing SINE/Alu and LINE/L1 elements, respectively (Fig. 5a). Outside of benchmark blacklist regions (Supplementary Fig. S14a), SV call set in NanoStrand-seq showed a precision of 83.60% (2952/3531) for deletions and 87.01% (4448/5112) for insertions, relative to the bulk PB-CCS dataset. Similarly, relative to the bulk ONT-UL dataset^43^, a precision of 94.53% (3338/3531) for deletions and 87.21% (4458/5112) for insertions were observed (Fig. 5b). Next, these SVs were resolved into two haplotypes, comprising 5184 deletions and 4667 insertions (Fig. 5c; Supplementary Fig. S14b, c). Extensively, we found that the majority of the SVs longer than 100 bp (62.25%, 3628/5828) contained repetitive elements as expected (Fig. 5e). Moreover, 14.17% of these SVs contained multi-copies or even multi-types of repetitive elements in a single SV event (termed complex SVs) (Supplementary Fig. S15a, b), illustrating the unique advantage of NanoStrand-seq in detecting complex SVs compared to short-read sequencing techniques. To evaluate the phasing accuracy of haplotype-specific SVs, we initially compared them against PB-CCS data, in which substantial SVs could be assigned to parental origin through SNP linkage within haplotype blocks. We demonstrated that among all haplotype-specific SVs defined in NanoStrand-seq, up to 87.61% of hetSVs can be validated by PB-CCS long-reads for parental origin based on the nearest hetSNPs annotated by GIAB, and 97.61% of which were consistent with PB-CCS reads. 12.42% of hetSVs still exceeded the phasing capacity of the PB-CCS approach (Fig. 5d). In addition to this, we phased the PB-CCS SV call sets by the haplotype-tagged PB-CCS reads, which showed high consistency with NanoStrand-seq SV call sets (99.68%) (Fig. 3f; Supplementary Fig. S14c). We chose 62 of the phased SVs and successfully validated 98.4% (61/62) of them through inspection of the linked hetSNP and hetSV edges by genomic PCR coupled with Sanger sequencing (Supplementary Fig. S16 and Table S5), highlighting the high performance of SV phasing using NanoStrand-seq.

Specifically, analysis of distance distribution between hetSVs and their nearest hetSNPs revealed that the majority of SVs located within 2 kb from their nearest hetSNPs. 381 of SVs were more than 2 kb away, with 61 of these SVs greater than 10 kb away from the nearest hetSNPs (Fig. 5f). Among these 61 SVs, 42.6% (26/61) of them occurred within gene body regions (23 within introns and 3 within untranslated regions), 26.2% (16/61) of them located within 100 kb from the nearest genes, and 31.2% (19/61) of them were located over 100 kb away from the nearest genes. For the current SV phasing approach, even the short-read-based Strand-seq analysis integrated with long-read-based approach, the distance (between hetSV and its nearest hetSNP) longer than the sequencing reads themselves would confound the haplotype phasing of SVs. For example, NanoStrand-seq could directly phase an SV with the nearest hetSNPs 14 kb away, while PB-CCS reads failed to phase it (Supplementary Fig. S17). We further successfully verified another 9 SVs that were over 10 kb away from the nearest hetSNPs using long DNA fragment PCR coupled with Nanopore sequencing. All the cases were verified in line with expectations (Supplementary Fig. S18 and Table S6). In the specific example in Supplementary Fig. S18g, only one haplotype was selectively enriched during PCR due to a length difference of 7331 bp between these two haplotypes, illustrating that NanoStrand-seq was particularly efficient in direct phasing of SVs located in long genomic regions of homozygosity.

### Haplotype-resolved genome assembly through the integration of PacBio HiFi reads and NanoStrand-seq

**Fig. 6:**
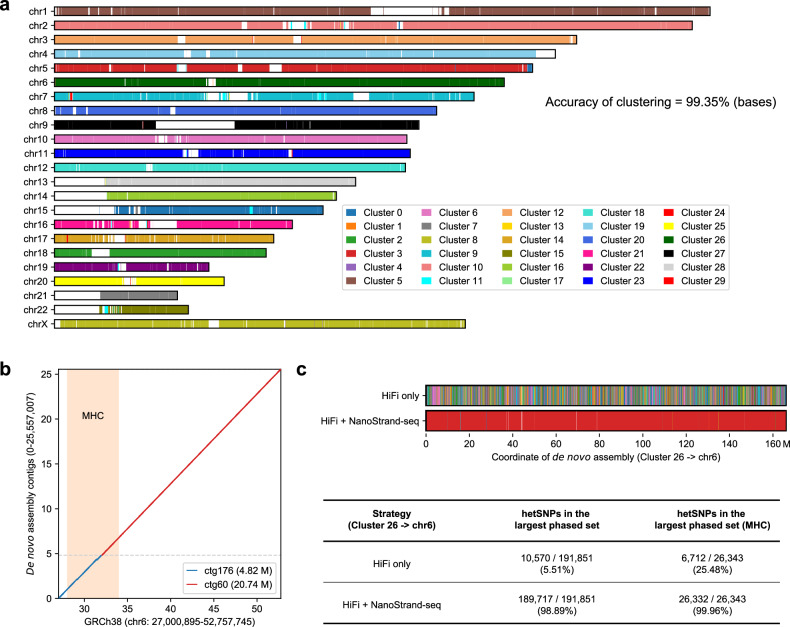
Haplotype-resolved de novo assembly by the combination of bulk HiFi long-read data and single-cell NanoStrand-seq data. **a** Squashed contigs clustered through NanoStrand-seq data were mapped against GRCh38. Each color represents contigs clustered in the same cluster. Ideally, each chromosome should only be marked with one color. **b** MHC assemblies were compared with GRCh38. Two contigs spanning 95.71% of the MHC region were displayed. **c** Comparison of phased haplotype blocks derived from only HiFi data and the combination of HiFi data with NanoStrand-seq data shown for cluster 26 (the vast majority of contigs in this cluster were mapped to chromosome 6) and MHC region. Each color represents a phased haplotype block; due to the numerous blocks, we represented them using only 10 distinguishable colors, with the largest haplotype block colored in red.

In addition, we also explored whether NanoStrand-seq enabled the accurate phasing of de novo assemblies produced by PacBio HiFi data (at depth of 28.15×, accession number: PRJNA540705). Initially, we created squashed de novo genome assemblies at the contig level by wtdbg2^44^, followed by contig clustering^23^. In detail, we filtered out contigs that were shorter than 100 kb, aligned 132 NanoStrand-seq libraries (with the same number as Strand-seq used in previous study^27^) to these remaining squashed contigs, and divided these contigs into 30 clusters by directional information. To evaluate the performance of clustering, we aligned these clustered contigs to the GRCh38 reference genome. The results showed that within 30 clusters, the vast majority of contigs in 26 clusters were individually mapped to the same chromosome, presenting that 99.35% of the total length of all clustered contigs were correctly placed into their respective chromosome origins (Fig. 6a). We then aligned PacBio HiFi reads against these squashed contigs and de novo called hetSNPs using NanoCaller^45^, and phased these hetSNPs through NanoStrand-seq. Subsequently, we converted the cluster-based hetSNPs to chromosome-based hetSNPs. The results showed that we retrieved 90.67% of hetSNPs relative to GIAB, and maintained a low overall Hamming error rate of 0.34% (99.66% of accuracy rate), comparable to Strand-seq under the same library number. Moreover, NanoStrand-seq streamlined MHC region assembly with only two contigs (Fig. 6b). 99.96% (26,332/26,343) of hetSNPs (detected by PacBio HiFi reads) within MHC region were assigned in the largest phase block (Fig. 6c), which was much higher than only PacBio HiFi reads (25.48%, 6712/26,343). In terms of Cluster 26 (largely corresponding to Chr6), the combination of NanoStrand-seq and PacBio HiFi data allocated 98.89% of hetSNPs to the largest phase block, while only 5.51% of hetSNPs were allocated to the largest phase block generated by localized phasing using PacBio HiFi data. These results clearly highlighted the advantage of NanoStrand-seq for haplotype-resolved genome assembly at whole-chromosome scale.

### Flexibility of NanoStrand-seq method

**Fig. 7:**
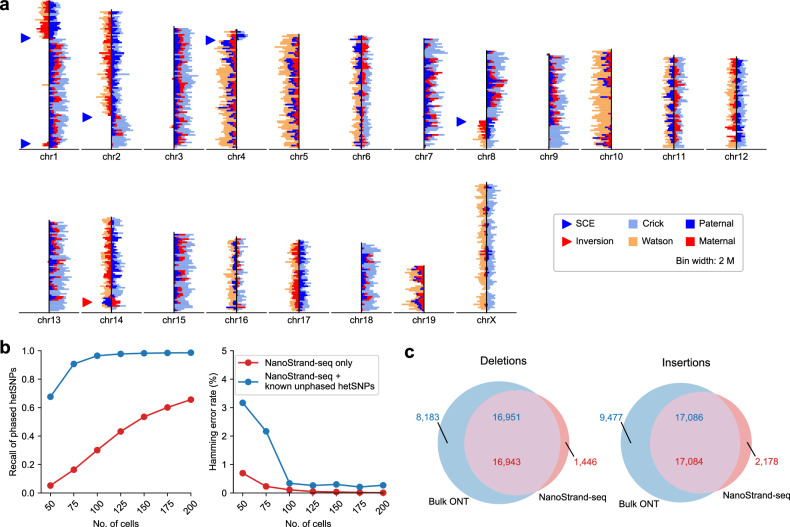
The performance of NanoStrand-seq with primary mouse cells. **a** Representative ideogram plot of a NanoStrand-seq library for primary mouse cells distinguishing three possible template strand inheritance patterns (WW, CC, CW) and visible SCEs and inversion events. Directional sequencing reads were aligned to the mm10 reference genome and read counts were plotted as horizontal lines for each chromosome. **b** The recall rate and Hamming error rate of de novo phasing hetSNPs by NanoStrand-seq only and the strategy in which NanoStrand-seq was combined with known hetSNPs (from MGP) that linked in the same reads with hetSNPs called by NanoStrand-seq. **c** The consistency of deletion and insertion events between NanoStrand-seq and bulk DBA data derived from ONT sequencing.

Finally, to determine whether the NanoStrand-seq workflow could still work well in other scenarios, we applied it to primary mouse endometrial epithelial cells and embryonic fibroblasts. These cells were isolated from F1 hybrid mice B6D2F1 (a cross between female C57BL/6J [B6] and male DBA/2NCrl [DBA]). We obtained 206 individual cells that passed the quality control (with ≥ 70,000 unique reads and < 5% background) (Fig. 7a; Supplementary Fig. S19a). The results showed barely GC bias (Supplementary Fig. S19b), with an average sequencing depth of 0.22× for each single cell. We randomly selected 200 cells for further analysis. Because the mm10 mouse reference genome (GRCm38) was built on the C57BL strain, SNPs and SVs identified from F1 hybrid mouse cells were theoretically derived from DBA. By comparing haplotype-resolved SNPs with the variants from MGP^46^, we identified ~3.66 million potential hetSNPs, maintained the precision of genotyping up to 97.49%, with the Hamming error rate as low as 0.01% (Supplementary Table S7). Similarly, using known hetSNPs derived from deep sequencing applied on the NGS platform, as few as 100 NanoStrand-seq libraries exhibited 96.48% of recall rate and 0.34% of Hamming error rate (Fig. 7b). In addition, 18,435 heterozygous deletions and 19,293 heterozygous insertions were efficiently detected and phased. 92.06% of deletions and 88.84% of insertions detected in NanoStrand-seq were concordant with the fully deep-sequenced bulk DBA ONT data (Fig. 7c)^47^. Furthermore, we reconstructed a full spectrum of the haplotype-specific SNPs and SVs. The distribution of all genetic variations at the whole-chromosome level exclusively presented in a single haplotype indicated the high phasing accuracy of our approach. Of interest, we observed that the distribution patterns of SNPs and SVs were similar to each other (Supplementary Fig. S19c). Together, these results showed that NanoStrand-seq was applicable to various cell types, similar to Strand-seq. Both methods can be used not only for immortalized cell lines but also for primary cultured cells with limited division potentials.

## Discussion

Collectively, we developed a novel technology, named NanoStrand-seq, which leveraged SMS platforms to perform simultaneous de novo calling and phasing of both SNPs and SVs directly at the whole-chromosome level. Note that the estimated base accuracy of NanoStrand-seq libraries was 99.21%, significantly lower than that of the NGS platform. However, in the subsequent analysis, the duplicate reads were incorporated. With support from multiple reads and multiple cells, we successfully obtained highly accurate phasing information of genetic variations at the whole-chromosome scale. In detail, we observed that NanoStrand-seq exhibited comparable phasing accuracy to short-read-based Strand-seq at high-confidence genomic regions annotated by GIAB. De novo calling and phasing SNPs in highly polymorphic genomic regions, such as medically important and hyper-polymorphic MHC region further emphasized the robustness of the NanoStrand-seq technique. Currently, accurate annotation and phasing information of SNPs within high-complexity genomic regions outside the high-confidence regions of GIAB has yet to be established. We demonstrated that NanoStrand-seq enabled calling and phasing of SNPs outside of GIAB-defined high-confidence genomic regions, particularly in regions rich with high-density gene clusters and highly polymorphic loci. The phasing accuracy of these SNPs was validated by various datasets. Notably, these datasets either lacked phase information or had limited calling sensitivity, whereas our method detected a significant number of SNPs with haplotype phasing information. This indicated that our method made a significant contribution to the calling and phasing of SNPs in these challenging genomic regions. Apart from that, similar to short-read-based Strand-seq, when combined with PB-CCS data or other long-read sequencing data, NanoStrand-seq was able to construct a high-quality haplotype-resolved de novo assembly of human genome.

A more distinguished advantage of the NanoStrand-seq was that it significantly extended the performance of the short-read-based Strand-seq approach, achieving efficient and reliable direct calling and phasing of SVs. In particular, NanoStrand-seq enabled a direct calling and phasing of complex SVs that were made up of multiple repetitive elements in a single SV event. Remarkably, NanoStrand-seq presented a significant advantage in direct phasing of SVs that located within long genome regions of homozygosity, surpassing other long-read-based techniques even when integrated with short-read-based Strand-seq. Furthermore, we demonstrated its applicability in primary cultured cells with similar performance, suggesting that NanoStrand-seq could adapt to diverse individuals or cell types. More importantly, we developed distinct experimental protocols and novel bioinformatic analysis pipelines, such as a de novo haplotype-specific SNP calling pipeline, specifically tailored for the NanoStrand-seq technique.

Future effort to increase read length and reduce sequencing error rate would further improve the power of NanoStrand-seq. In addition, our approach relied on an existing reference genome, limiting the detection of new complex SVs relative to the reference genome. Accordingly, there is a need to extend this method to obtain even longer DNA fragments to generate continuous sequences using assembly algorithms. This will greatly improve the completeness of the haplotype-resolved SVs, and contribute to the detection of missed sequences and haplotypes that are not present in the current reference genome.

The cost of NanoStrand-seq was ~US$14 per library excluding sequencing, similar to Strand-seq (US$13). Still, NanoStrand-seq had innate cost-intensive limitations primarily due to the high expense of the sequencing flow cells in the ONT platform. Currently, the ONT sequencing cost for each library is US$13, generating 1 Gb sequencing data for each single cell in this study. However, we believe that the sequencing cost tends to decrease over time. To date, NanoStrand-seq is the only method capable of phasing both SNPs and a wide range of SVs in diploid cells directly at whole-chromosome scale. It is also feasible to use NanoStrand-seq to phase polyploid genomes. For example, in triploid or tetraploid genomes, when the template strand direction of one homolog is opposite to that of the remaining homologs, we should be able to unambiguously figure out the combination of all genetic variants on this homolog (Supplementary Fig. S20). The underlying principle of this technique mirrors that of Strand-seq^48^. Both methods were superior to methods that relied on haploid somatic cells or relied on haploid gametes that were only abundantly available from male individuals. They were also superior to localized phasing methods. Consequently, we foresee that NanoStrand-seq opens up new avenues for complete genetic variant phasing at the whole-chromosome scale, and provides a powerful tool for future haplotype-related genetic studies.

## Materials and methods

### Cell culture

Cells were maintained at 37 °C in a 5% CO_2_ atmosphere. GM12878 (HG001) cell line was purchased from the Coriell Institute and confirmed using STR authentication. Cells were grown in RPMI1640 (Gibco; 11875093) medium containing 15% fetal bovine serum (Gibco; 26140079), 100 U/mL penicillin, 100 U/mL streptomycin (Gibco; 15140122). Primary mouse embryonic fibroblasts and primary mouse endometrial epithelial cells were isolated from C57BL/6J female and DBA/2NCrl male hybrid mice. These cells were maintained in Dulbecco’s modified Eagle’s medium (DMEM, Gibco; 11995040) consisting of 15% fetal bovine serum and 1% L-glutamine (Gibco; 2503081). Animal experiments were performed according to the guidelines of the Institutional Animal Care and the Ethics Committee of Peking University. The research license number is LSC-TangFC-4.

### The procedure of NanoStrand-seq

#### Preparation of single-cell suspension

In brief, cells with a confluency of 50%–60% were treated with 10 µM (final) RO-3306 (Sigma; SML0569) for 4 h to synchronize cells at G2/M transition. Then cells were released into BrdU (Sigma; B9285) contained culture medium for the indicated concentrations based on cell types. In this study, the GM12878 was pulsed with 40 μM (final) BrdU for 24 h, and primary mouse endometrial epithelial cells and embryonic fibroblasts were pulsed with 80 μM (final) BrdU for 24 h. Cells were then washed twice with cold PBS and dissociated in nuclei isolation buffer (100 mM Tris-HCl, pH 7.4, 150 mM NaCl, 1 mM CaCl_2_, 0.5 mM MgCl_2_, 0.1% (v/v) NP-40 and 2% bovine serum albumin) for 10 min on ice followed by addition of Hoechst 33258 (Sigma; 94403) and propidium iodide (PI, Sigma; P4170) at final concentrations of 10 µg/mL. The PI signal indicated the cell cycle stage, and the Hoechst signal indicated whether BrdU had been absorbed into the cell nuclei. This is because BrdU absorbs the Hoechst signal, resulting in the BrdU-labeled cell nuclei displaying half of the Hoechst signal intensity compared to BrdU-unlabeled nuclei^26^. Cells with significantly half decreased Hoechst 33258 signal at the G1 phase were further chosen (for gating details, see Supplementary Fig. S1a). These cells were sorted directly into 2.5 μL lysis buffer (2 mM Tris-EDTA, pH 8.0, 1 mg/mL Qiagen protease (Qiagen; 19155), 0.3% (v/v) Triton X-100, 20 mM KCl) and lysed at 50 °C for 3 h, 70 °C for 30 min to remove proteins from DNA. Lysed cells could be stored at −80 °C if not immediately amplified (until further processing).

#### Preparation of transposon complex

Before cell handling, both DNA oligos (ME: 5′-phos-CTGTCTCTTATACACATCT-NH_2_-3′, S5: 5′-TCGTCGGCAGCGTCAGATGTGTATAAGAGACAG-3′, purification: HPLC) were dissolved in PCR-grade Ultrapure distilled water to a final concentration of 100 µM and were annealed to be adaptor at a final concentration of 50 µM. Tn5 transposase was purchased from Vazyme and the transposon complex was assembled following the manufacturer’s protocol instructions (Vazyme; S111-01). In detail, the transposon complex was assembled by the addition of 10 μL Tagment Enzyme (2 μg/μL), 7 μL adaptor (50 μM) and 33 μL Coupling Buffer, performed at 55 °C for 10 min. Note that the length of DNA fragments was transposon concentration-dependent. Herein, we recommended a 1:5000 dilution of the transposon complex using the storage buffer composed of 50% (v/v) glycerol (Sigma; G5516), 50 mM HEPES, pH 7.2, 100 mM NaCl, 0.1 mM EDTA, 1 mM DTT, 0.1% (v/v) Triton X-100. Diluted transposon complex was aliquoted for single uses and stored at −80 °C.

#### Tagmentation and gap-filling

Single cell was tagmentated by the addition of 2.5 μL transposition mix including 1 μL 5× TAPS_PEG8K (50 mM TAPS-NaOH (or KOH), pH 8.3 (room temperature), 25 mM MgCl_2_, 40% PEG8K), 1 μL transposon complex (1:5000 diluted) and 0.5 μL H_2_O. Tagmentation was performed at 55 °C for 10 min followed by adding 1 μL stop buffer composed of 0.25% SDS, and incubated at 55 °C for 10 min to denature transposases. Then 1.25 μL 20% (v/v) Tween 20 was added to quench remaining/persisting SDS and incubated for 5 min at room temperature. Next, 0.5 μL Bst 2.0 DNA Polymerase (New England Biolabs; M0537), 1 μL 10× Isothermal Amplification Buffer, 0.5 μL 10 mM dNTPs, and 0.75 μL H_2_O were subsequently added to perform gap-filling on tagmented DNA, with an incubation at 65 °C for 30 min. Further, the gap-filled DNA fragments were purified once with 0.8× AMPure XP Beads (Beckman; A63882), and eluted with 9 μL H_2_O.

#### Nick the nascent DNA by UV treatment

The purified blunt-end DNA fragments were added with 1 μL Hoechst33258 (1 mg/mL) and transferred to a UVP crosslinker (Analytikjena; CL-1000L) equipped with 365 nm longwave UV lamp, followed by treatment with 3.0 × 10^3 ^J/m^2^ exposure to nick the nascent DNA incorporated with BrdU.

#### Strand tagging

The following strand tagging procedures were conducted as follows: first-strand tagging was performed by addition of 13.5 μL Strand Tagging Mix 1 containing 10 μL 2× Gflex PCR Buffer (Mg^2+^, dNTP plus), 0.2 μL Tks Gflex DNA Polymerase (TAKARA; R060B), and 200 nM first-strand PCR primer which contained 24-bp barcode (5′-ACACTCTTTCCCTACACGACGCTCNNNNNNNNNNNNNNNNNNNNNNNNTCGTCGGCAGCGTCAGATGTG-3′) (see Supplementary Table S1 for 1st barcode primers). This reaction was performed at 94 °C for 2 min, 62 °C for 5 min, and 68 °C for 10 min, followed by the addition of 2 μL ExoI (NEB; M0293) incubating at 37 °C for 30 min, 80 °C for 20 min to digest the residue primers. Then first-strand tagged products were purified with 0.8× AMPure XP Beads once and eluted with 10 μL H_2_O.

Second-strand tagging was performed by the addition of 13.5 μL Strand Tagging Mix 2 containing 10 μL 2× Gflex PCR Buffer (Mg^2+^, dNTP plus), 0.2 μL Tks Gflex DNA Polymerase, and 200 nM second-strand PCR primer (see Supplementary Table S1 for 2nd barcode primers) whose barcode was distinct from the first-strand tagging primers above, while the PCR program was the same as first-strand tagging procedure. After that, second-strand tagging primer was digested by the addition of 2 μL ExoI and incubated at 37 °C for 30 min, and 80 °C for 20 min. Then tagged products were purified with 0.8× AMPure XP Beads once, and eluted with 11 μL H_2_O.

#### Amplification of single-cell genomic DNA

Strand tagging products were added with 1.5 μL 20 μM Amplification primer (5′-ACACTCTTTCCCTACACGACGCTC-3′) and 0.3 μL Tks Gflex DNA Polymerase (1.25 U/μL), 12.5 μL 2× Gflex PCR Buffer (Mg^2+^, dNTP plus) to a total of 25 μL. The reaction was performed at 94 °C, 3 min, 21 cycles of 98 °C for 15 s, 62 °C for 30 s, and 68 °C for 8 min; followed by 68 °C for 10 min and 4 °C hold on a thermal cycler. Next, the amplification products were purified with 0.8× AMPure XP Beads once and then quantified by Qubit double-stranded DNA High-sensitivity assay following the manufacturer’s instructions (Thermo Fisher; Q32851). Finally, purified amplification products with different pairs of first-strand and second-strand tagging barcodes were then pooled together at an equal amount. As a lower percentage of barcodes in shorter DNA fragments (typically <1 kb), we purified the pooled amplicons with 0.7× AMPure XP Beads twice for the selection of the long-length fragment. A representative Bioanalyzer trace was shown in Supplementary Fig. S1b. A total of ~1 μg purified amplicon products were used for further library construction (adaptor in SQK-LSK109), and sequenced on Oxford Nanopore PromethION 48 (Oxford Nanopore Technologies; R9.4.1). In this study, we sequenced a total of 826 individual cells (with 9 flow cells) for GM12878 cells and 455 individual cells (with 5 flow cells) for mouse cells.

### Demultiplex of long reads of Nanopore sequencing platform

The long reads produced by one PromethION flow cell (Oxford Nanopore) originated from dozens of cells, which can be distinguished by different 1st barcode and 2nd barcode combinations. Firstly, we extracted 200-bp of sequences at the head and tail ends of reads (no more than half the length of reads) and aligned these sequences against 1st barcode sequences. We identified the best hit through minimal Levenshtein distance and recorded the location (head or tail ends) and alignment orientation (forward or reverse). Then, we repeated the process for 2nd barcode sequences. According to our statistical results (Supplementary Fig. S2d), we discarded the hits that edit distance > 5. The origin of reads was determined by the location and orientation of the barcode combination. The template strand reads were expected to contain a forward 2nd barcode sequence at the head of reads and a reverse 1st barcode sequence at the tail of reads. In contrast, the non-template strand reads were expected to contain a forward 1st barcode sequence at the head of reads and a reverse 2nd barcode sequence at the tail of reads (Supplementary Fig. S2a). Next, we converted the non-template strand reads into template strand reads by reverse complement. Then, the barcode sequences were removed. Finally, the reads with identical barcode combinations were separated into the same FASTQ file.

### Genome mapping and post-mapping data processing

Firstly, under the edit distance ≤ 8, we searched the linker sequences (TCGTCGGCAGCGTCAGATGTGTATAAGAGACAG, 33 bp) within 40 bp sequences of both head and tail ends of the single-cell assigned reads and we removed the linker sequences. The reads without linker sequences were discarded. We searched the linker sequences in the trimmed reads again (edit distance ≤ 8) to remove potential chimeric reads. Secondly, the trimmed reads were aligned to the *Homo sapiens* genome (GRCh38) or *Mus musculus* genome (GRCm38) with minimap2 (v2.24-r1122)^49^ (command: “minimap2 -ax map-ont –MD -R”). The unmapped, secondary, supplementary, mitochondria, and low-quality (MAPQ < 30) alignments were removed by samtools (v1.16.1)^50^. Thirdly, the reads with identical strands and similar edges (the difference between each coordinate of the start and end of reads was less than 20 bp) were marked as PCR duplicates. Finally, we annotated the parental origin for the alignments according to the phased hetSNPs. For each alignment that overlaps with one or more hetSNPs, we identified the alleles at the hetSNPs position and determined paternal or maternal origin. Only the alignments that solely contain paternal or maternal alleles (unambiguous) were annotated. The phased hetSNPs of GM12878 were downloaded from the GIAB database (https://www.nist.gov/programs-projects/genome-bottle, NISTv4.2.1), and the phased hetSNPs of mouse cells were obtained from MGP database (https://www.sanger.ac.uk/data/mouse-genomes-project/)^46^.

### Quality control of NanoStrand-seq libraries

The background could be recognized as noise and it was inversely correlated to the phasing accuracy of hetSNPs. Therefore, we rigorously filtered the cells and read counts to minimize the impact of background noise (Supplementary Fig. S3). For short-read-based Strand-seq (Strand-seq and OP-Strand-seq) and NanoStrand-seq, both the evaluation and criteria of background were the same. In detail, we calculated the read number that mapped to the forward strand (Crick) and reverse strand (Watson), respectively, for each chromosome. We used the proportion of Crick reads (P_c_) to measure the strand distribution pattern for each chromosome. Then we calculated the background for each chromosome in each cell as follows:

$${{Background}}_{{chrom}}=\frac{{MIN}\left({N}_{{chrom},{crick}},{N}_{{chrom},{watson}}\right)}{{N}_{{chrom},{crick}}+{N}_{{chrom},{watson}}}$$

We calculated the mean value in four minimum backgrounds within the autosomes and denoted it as the background noise of each cell both in short-read-based Strand-seq and NanoStrand-seq. For GM12878, libraries with ≥ 80,000 unique reads and < 5% background were retained (364/826). We randomly selected 350 cells for the downstream analysis. While, for primary mouse endometrial epithelial cells and embryonic fibroblast cells, libraries with ≥ 70,000 unique reads and < 5% background were retained (206/455). We randomly selected 200 cells for the downstream analysis.

### Identification of putative inversion events

To generate the CC (Crick-Crick) composite file for a certain chromosome, we chosed cells with potential CC pattern (P_c_ > 0.8) or WW (Watson-Watson) pattern (P_c_ < 0.2). Then, the read direction of the WW chromosomes was switched and turned into CC pattern^51^. Next, we binned the chromosome to a width of 1 Mb and calculated the P_c_ of each bin. We clustered the cells according to the P_c_ value of all bins. The excepted P_c_ was calculated for each bin. We extracted the continuous bins that showed a similar pattern to the expected P_c_ for each cell and marked them as pure CC regions (Supplementary Fig. S5). The reads overlapping with pure CC regions were merged to build a CC composite file. To automatically identify putative inversion regions, we scanned the CC composite file of the whole chromosome, selected the regions with continuous coverage of Watson reads, and counted the read counts of Crick and Watson for those regions. The regions with less than 20 reads (including both Crick and Watson reads) were filtered out. The remaining regions were classified by the proportion of Watson (P_w_) reads: (1) if the P_w_ > 0.9, the region was a putative homozygous inversion; (2) if the P_w_ > 0.4 and P_w_ < 0.6, the region was a putative heterozygous inversion; (3) if the P_w_ < 0.1, the region will be filtered out; (4) otherwise, the region was an ungenotyped putative inversion.

### Pipeline of de novo calling of haplotype-resolved genome

Firstly, we merged all cell alignments, and de novo called SNPs by using NanoCaller (v1.0.0)^45^ with parameter: --snp_model ONT-HG001 --indel_model ONT-HG001. Then we constructed a blacklist region that contains too many reads^51^. The reads that overlap with the blacklist region were discarded in the downstream pipeline. For a certain chromosome of single cell, we screened out the WC (Watson-Crick or Crick-Watson) reads, and determined the allele for Crick reads and Watson reads using hetSNPs. For any two cells, we compared their allele between Crick and Watson pairs and determined whether they should be merged as the CC-WW (log_2_FC > 0) pattern or CW-WC (log_2_FC < 0) pattern. The log_2_FC of all pairwise comparisons were clustered and the cells were significantly separated into two clusters. The Crick reads of cluster 1 were merged with Watson reads of cluster 2, and vice versa. Thus, we constructed two bam files that each contained reads from only one haplotype. Finally, we de novo called SNPs from haplotype-resolved bam files by analyzing the consensus alleles for each haplotype (Fig. 3a; Supplementary Figs. S8 and S9).

To improve the recall rate of hetSNP, we performed haplotype reconstruction again, which we called round 2 haplotype reconstruction. In round 2 haplotype reconstruction, we annotated the reads by de novo called and phased hetSNPs and separated the reads into haplotype 1 or haplotype 2 reads (Supplementary Fig. S10a). This step rescued more reads for the reconstruction of haplotype-resolved bam files. The phased hetSNPs were switched the haplotype information when hetSNPs were located within putative homozygous inversion regions (Fig. 3a).

### De novo assembly by the integration of Pacbio HiFi data with NanoStrand-seq data

We downloaded the PacBio HiFi data for GM12878 cells from PRJNA540705. The HiFi reads were assembled into contigs using wtdbg2 with parameters “-x ccs -g 3.1g”. The reads of 134 NanoStrand-seq libraries were mapped to the consensus contigs with minimap2 using default parameters. The process of filtering alignment and duplicate reads labeling was the same as the step of genome mapping as described above. We constructed a matrix, in which each row represented a contig and each column represented a cell. The cell value was (Crick – Watson)/(Crick + Watson). The contigs shorter than 100 kb were filtered. Firstly, the absolute value of the matrix was clustered by the AgglomerativeClustering function of the python sklearn package (n_clusters=30, linkage=“complete”, compute_distances=True). The contigs in the same cluster (same chromosome) were clustered again by the raw cell value of the matrix and were divided into two sub-clusters. To generate cluster sequence, the contigs of the one sub-cluster were reverse complement and concatenated with the contigs of another sub-cluster with a 1-kb blank gap. The HiFi reads were mapped to the cluster sequence and we de novo called hetSNPs as anchors. The NanoStrand-seq reads were mapped to the cluster sequence and called the haplotype-resolved genome as described above using the anchors of hetSNPs. The coordinate of the phased hetSNPs was cluster-based and was converted into chromosome-based according to the mapping of contig sequences to the reference genome (GRCh38).

### Identification and phasing of SVs

We called SV from merged bam files (from 350 NanoStrand-seq cells) using cuteSV (v2.0.1)^52^ with default parameters. The SVs inside blacklist regions were removed. The definition of blacklist regions was defined as Zook group^53^. In brief, SVs within 1 kb of adjacent SV and those longer than 10 kb, regions within low genomic coverage (< 10×) or high genomic coverage (> 80×) in both PB-CCS and ONT-UL datasets were excluded. These complex regions often lacked reliable SV information or contained multiple SVs. Besides, challenging genetic contexts were also excluded, including difficult regions longer than 200 bp, and tandem regions longer than 200 bp, which were extracted from GRCh38-stratification call set (Supplementary Fig. S14a). We obtained the annotation of repetitive elements from RepeatMasker and counted the repetitive elements that overlap with SVs. We quantified the SVs using haplotype bam files and determined whether the SV exists or not in any certain haplotype. In detail, the homozygous SVs were supported by both haplotypes, and the heterozygous SVs were supported by one haplotype and opposed by another haplotype. The haplotype information of phased heterozygous SVs was switched when they located at putative homozygous inversion regions.

We directly compared the detection performance of SVs between NanoStrand-seq and Strand-seq. Direct detection of SVs by Strand-seq was addressed by Ashley and colleagues, in which, they performed Strand-seq to transformed epithelial cells and patient-derived leukemic samples, with 80% of samples being diploid. Given that HG001 is also a diploid cell line, we believe the number of SVs in the genomes are comparable across different human diploid cell lines. Thus it is feasible to directly compare these two technologies to illustrate the capacity of SV detection, primarily focusing on deletions and insertions. We observed that Strand-seq detected various types of SVs in the range of dozens, while NanoStrand-seq could detect 5185 deletions and 4667 insertions in a diploid cell line (Supplementary Table S3), demonstrating that NanoStrand-seq largely expanded the detection range and quantity of genetic variations.

### Bulk DNA extraction and validation of structure variations

Genomic DNA (gDNA) of GM12878 cells was extracted using the QIAGEN DNeasy Blood and Tissue Kit following the manual’s instructions (QIAGEN, 69504).

We employed a combination of PCR and Sanger sequencing to validate heterozygous SVs located within 500 bp away from the nearest hetSNPs. PCR primers were designed to ensure that the amplicons simultaneously covered both the SV and corresponding neighboring hetSNP (Supplementary Table S5). PCR was performed immediately by the addition of 5 μL of 2× Gflex PCR Buffer (Mg^2+^, dNTP plus), 0.2 μL of Tks Gflex DNA Polymerase (1.25 U/μL), 4 ng gDNA, 0.6 μL primers (10 μM each) and 4.15 μL of H_2_O. The PCR reactions were performed as follows: 94 °C for 1 min; 32 cycles of 98 °C for 10 s, 62 °C for 15 s, 68 °C for 1 min; 68 °C for 5 min; 4 °C hold. The PCR amplicons were subjected on a 0.8% agarose gel. Bands corresponding to both the wild-type alleles and mutant alleles from the gel were recovered and subsequently subjected to Sanger sequencing.

For validation of heterozygous SVs with the nearest hetSNPs located more than 10 kb away, PCR primers were also designed to simultaneously cover both the SV and the nearest hetSNP (Supplementary Table S6). PCR was performed immediately by the addition of 5 μL of 2× Gflex PCR Buffer (Mg^2+^, dNTP plus), 0.2 μL of Tks Gflex DNA Polymerase (1.25 U/μL), 4 ng gDNA, 1 μL primers (10 μM each) and 3.75 μL of H_2_O. The PCR reactions were performed as follows: 94 °C for 1 min; 32 cycles of 98 °C for 10 s, 62 °C for 15 s, 68 °C for 25 min; 68 °C for 30 min; 4 °C hold. Subsequently, the PCR amplicons were submitted to further library construction and sequencing on Oxford Nanopore PromethION 48.

### Supplementary information

Supplementary_Figures

Supplementary_Tables

## Data Availability

The data processing scripts and analysis workflows in this study are publicly available through the GitHub repository (https://github.com/Ckenen/NanoStrandseq-Project-Workstation). NanoStrand-seq libraries selected for this study have been submitted to the NCBI BioProject (https://www.ncbi.nlm.nih.gov/bioproject) under accession number PRJNA961850.

## References

[1] Tewhey R, Bansal V, Torkamani A, Topol EJ, Schork NJ (2011). The importance of phase information for human genomics. Nat. Rev. Genet..

[2] Judson R, Stephens JC, Windemuth A (2000). The predictive power of haplotypes in clinical response. Pharmacogenomics.

[3] Wu X (2002). p53 Genotypes and haplotypes associated with lung cancer susceptibility and ethnicity. J. Natl. Cancer Inst..

[4] Ebert P (2021). Haplotype-resolved diverse human genomes and integrated analysis of structural variation. Science.

[5] Falconer E (2012). DNA template strand sequencing of single-cells maps genomic rearrangements at high resolution. Nat. Methods.

[6] Porubský D (2016). Direct chromosome-length haplotyping by single-cell sequencing. Genome Res..

[7] Mahmoud M (2019). Structural variant calling: the long and the short of it. Genome Biol..

[8] Genomes Project C (2015). A global reference for human genetic variation. Nature.

[9] Logsdon GA, Vollger MR, Eichler EE (2020). Long-read human genome sequencing and its applications. Nat. Rev. Genet..

[10] Dubois F, Sidiropoulos N, Weischenfeldt J, Beroukhim R (2022). Structural variations in cancer and the 3D genome. Nat. Rev. Cancer.

[11] Soemedi R (2012). Contribution of global rare copy-number variants to the risk of sporadic congenital heart disease. Am. J. Hum. Genet..

[12] Elia J (2011). Genome-wide copy number variation study associates metabotropic glutamate receptor gene networks with attention deficit hyperactivity disorder. Nat. Genet..

[13] Browning SR, Browning BL (2011). Haplotype phasing: existing methods and new developments. Nat. Rev. Genet..

[14] Ge B (2009). Global patterns of cis variation in human cells revealed by high-density allelic expression analysis. Nat. Genet..

[15] Zhou Y, Leung AW, Ahmed SS, Lam TW, Luo R (2022). Duet: SNP-assisted structural variant calling and phasing using Oxford nanopore sequencing. BMC Bioinforma..

[16] Glusman G, Cox HC, Roach JC (2014). Whole-genome haplotyping approaches and genomic medicine. Genome Med..

[17] Mahmoud M, Doddapaneni H, Timp W, Sedlazeck FJ (2021). PRINCESS: comprehensive detection of haplotype resolved SNVs, SVs, and methylation. Genome Biol..

[18] Zheng GX (2016). Haplotyping germline and cancer genomes with high-throughput linked-read sequencing. Nat. Biotechnol..

[19] Zhang F (2017). Haplotype phasing of whole human genomes using bead-based barcode partitioning in a single tube. Nat. Biotechnol..

[20] Garg S (2021). Chromosome-scale, haplotype-resolved assembly of human genomes. Nat. Biotechnol..

[21] Garg S (2023). Towards routine chromosome-scale haplotype-resolved reconstruction in cancer genomics. Nat. Commun..

[22] Chaisson MJP (2019). Multi-platform discovery of haplotype-resolved structural variation in human genomes. Nat. Commun..

[23] Porubsky D (2021). Fully phased human genome assembly without parental data using single-cell strand sequencing and long reads. Nat. Biotechnol..

[24] Eichler EE, Clark RA, She X (2004). An assessment of the sequence gaps: unfinished business in a finished human genome. Nat. Rev. Genet..

[25] Gilissen C (2014). Genome sequencing identifies major causes of severe intellectual disability. Nature.

[26] Sanders AD, Falconer E, Hills M, Spierings DCJ, Lansdorp PM (2017). Single-cell template strand sequencing by Strand-seq enables the characterization of individual homologs. Nat. Protoc..

[27] Porubsky D (2017). Dense and accurate whole-chromosome haplotyping of individual genomes. Nat. Commun..

[28] Zhao Z (2022). STI PCR: an efficient method for amplification and de novo synthesis of long DNA sequences. Mol. Plant.

[29] Fan X (2021). SMOOTH-seq: single-cell genome sequencing of human cells on a third-generation sequencing platform. Genome Biol..

[30] Li W (2023). scNanoHi-C: a single-cell long-read concatemer sequencing method to reveal high-order chromatin structures within individual cells. Nat. Methods.

[31] Hanlon VCT (2022). Construction of Strand-seq libraries in open nanoliter arrays. Cell Rep. Methods.

[32] Piovesan A (2019). On the length, weight and GC content of the human genome. BMC Res. Notes.

[33] Sanders AD (2016). Characterizing polymorphic inversions in human genomes by single-cell sequencing. Genome Res..

[34] Porubsky D (2022). Recurrent inversion polymorphisms in humans associate with genetic instability and genomic disorders. Cell.

[35] Logsdon GA (2021). The structure, function and evolution of a complete human chromosome 8. Nature.

[36] Zook JM (2014). Integrating human sequence data sets provides a resource of benchmark SNP and indel genotype calls. Nat. Biotechnol..

[37] Zook JM (2019). An open resource for accurately benchmarking small variant and reference calls. Nat. Biotechnol..

[38] Horton R (2004). Gene map of the extended human MHC. Nat. Rev. Genet..

[39] Trowsdale J, Knight JC (2013). Major histocompatibility complex genomics and human disease. Annu. Rev. Genomics Hum. Genet..

[40] Choo SY (2007). The HLA system: genetics, immunology, clinical testing, and clinical implications. Yonsei Med. J..

[41] Chin CS (2020). A diploid assembly-based benchmark for variants in the major histocompatibility complex. Nat. Commun..

[42] Fairley S, Lowy-Gallego E, Perry E, Flicek P (2020). The International Genome Sample Resource (IGSR) collection of open human genomic variation resources. Nucleic Acids Res..

[43] Jain M (2018). Nanopore sequencing and assembly of a human genome with ultra-long reads. Nat. Biotechnol..

[44] Ruan J, Li H (2020). Fast and accurate long-read assembly with wtdbg2. Nat. Methods.

[45] Ahsan MU, Liu Q, Fang L, Wang K (2021). NanoCaller for accurate detection of SNPs and indels in difficult-to-map regions from long-read sequencing by haplotype-aware deep neural networks. Genome Biol..

[46] Keane TM (2011). Mouse genomic variation and its effect on phenotypes and gene regulation. Nature.

[47] Xie H (2023). Long-read-based single sperm genome sequencing for chromosome-wide haplotype phasing of both SNPs and SVs. Nucleic Acids Res..

[48] Sanders AD (2020). Single-cell analysis of structural variations and complex rearrangements with tri-channel processing. Nat. Biotechnol..

[49] Li H (2018). Minimap2: pairwise alignment for nucleotide sequences. Bioinformatics.

[50] Danecek P (2021). Twelve years of SAMtools and BCFtools. Gigascience.

[51] Hanlon VCT, Mattsson CA, Spierings DCJ, Guryev V, Lansdorp PM (2021). InvertypeR: Bayesian inversion genotyping with Strand-seq data. BMC Genomics.

[52] Jiang T (2020). Long-read-based human genomic structural variation detection with cuteSV. Genome Biol..

[53] Zook JM (2020). A robust benchmark for detection of germline large deletions and insertions. Nat. Biotechnol..

