# Supplementary material for: Simultaneous de novo calling and phasing of genetic variants at chromosome-scale using NanoStrand-seq
Source: Cell Discov. 2024 Jul 9;10:74. doi: 10.1038/s41421-024-00694-9 (PMC11231365; doi:10.1038/s41421-024-00694-9)
Supplement: Supplementary file 1 — Supplementary_Figures [file 41421_2024_694_MOESM1_ESM.pdf]

Supplementary Fig. S1

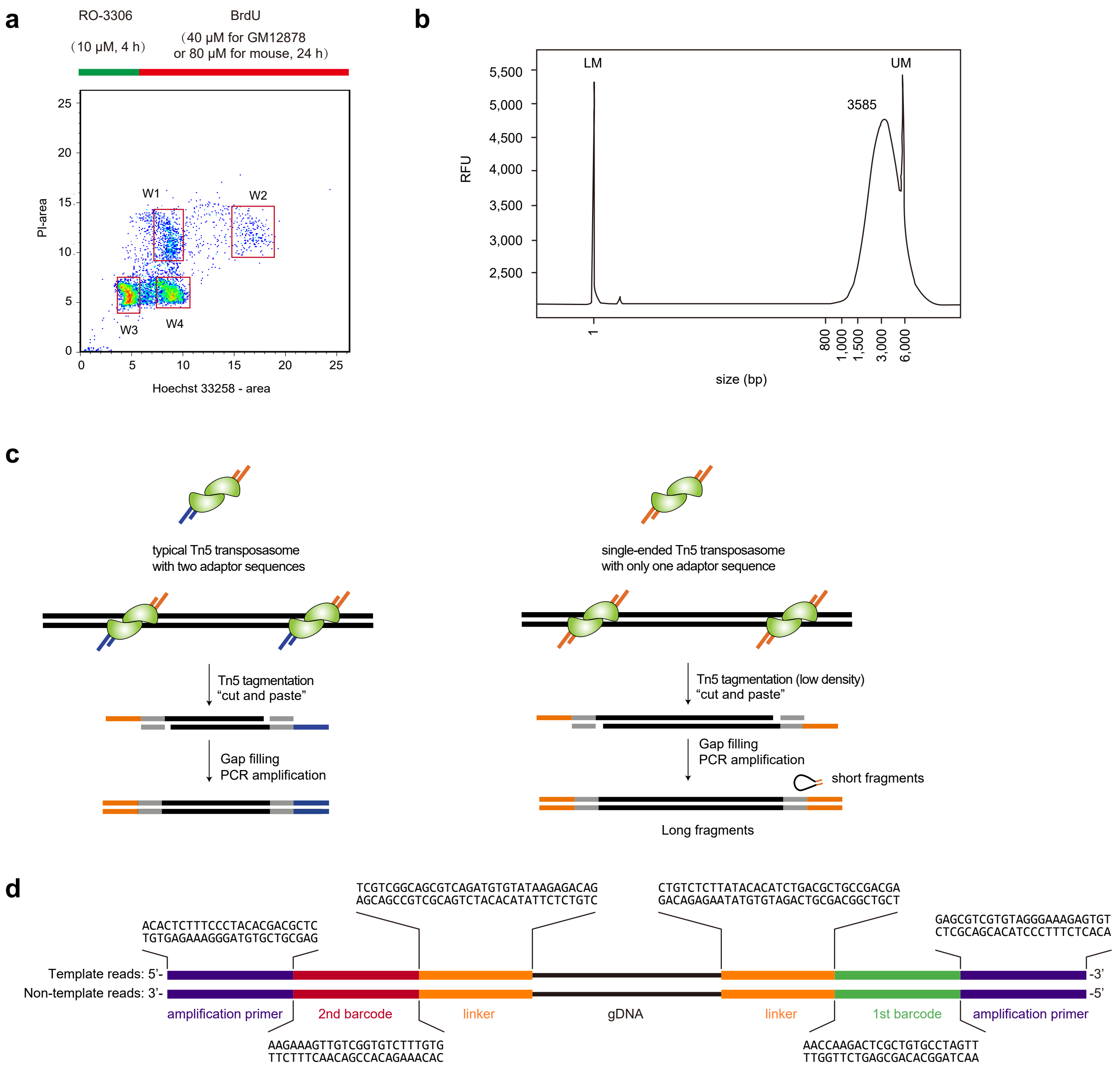

Supplementary information, Fig. S1 Characterization of NanoStrand-seq library in GM12878 cells.

a, Drug treatment and representative gating strategy for the BrdU-hemi-substituted G1 nuclei. W1 gate (high PI and low Hoechst fluorescence signal) represented BrdU-labeled parental cells that were currently in the G2 phase; W2 gate (high PI and high Hoechst fluorescence signal) represented BrdU-unlabeled parental cells that were currently in the G2 phase; W3 gate (low PI and low Hoechst fluorescence signal) represented daughter cells that were generated after one round of division with BrdU incorporation during the S phase; W4 gate (low PI and high Hoechst fluorescence signal) represented daughter cells that were generated after one round of division without BrdU incorporation during the S phase. **b,** Size distribution of an example NanoStrand-seq library measured by Fragment Analyzer. RFU, relative fluorescence units. LM, lower marker. UM, upper marker. **c,** Schematic diagram of transposition and PCR amplification for Tn5 transposase with two (left)[1] or one (right) adaptor sequence. **d,** Illustration of the library structure of NanoStrand-seq. Here, we only take one pair of barcode combinations as an example. The only difference among other libraries is the barcode combinations.

Supplementary Fig. S2

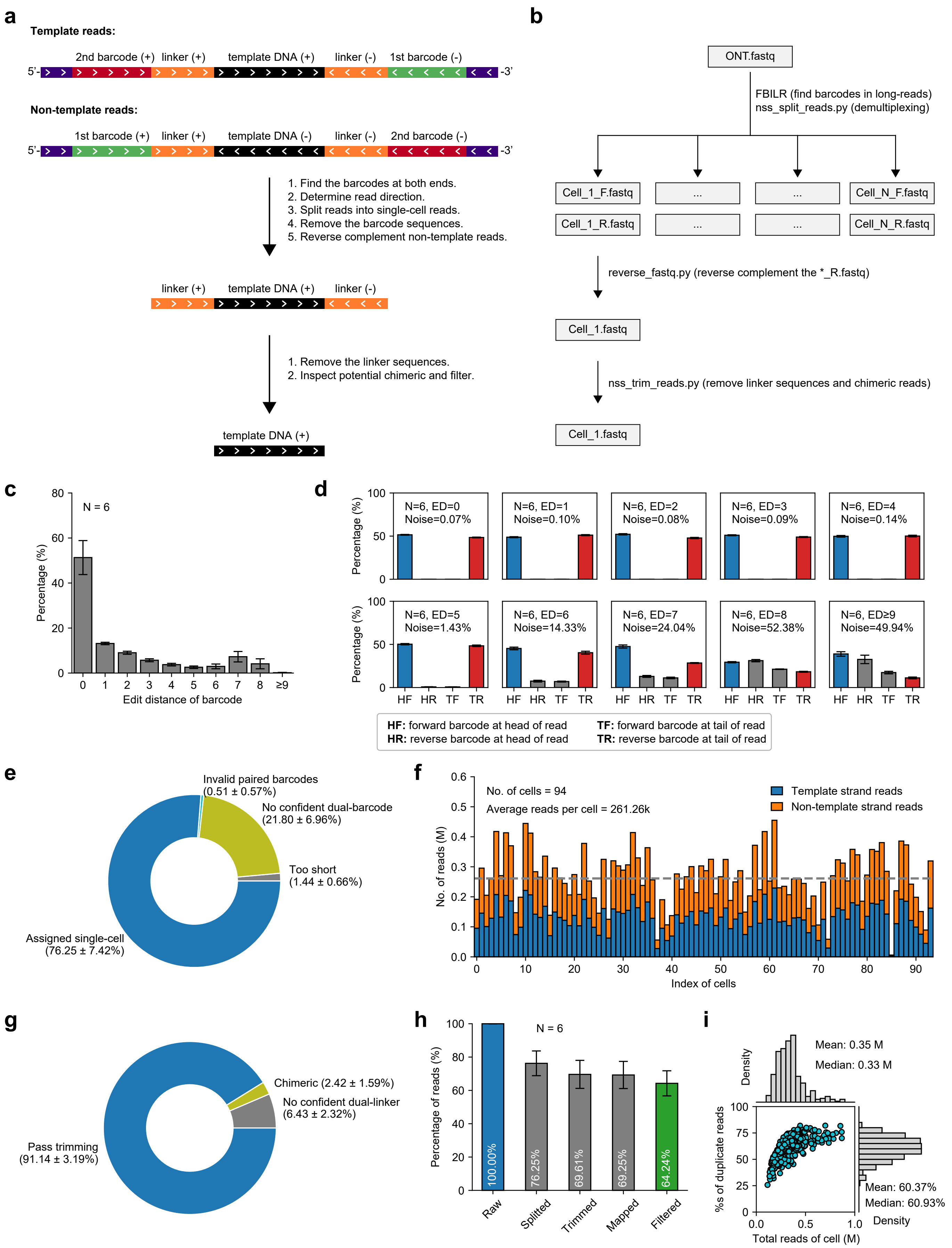

Supplementary information, Fig. S2 Data processing workflow and data quality of NanoStrand-seq.

a, Schematic showing the process of data splitting and cleaning of NanoStrand-seq. Chimeric reads were derived from the mis-ligation of different fragments. **b,** Detail workflow showing data splitting and cleaning of NanoStrand-seq. **c,** Percentage of reads obtained for barcodes at different editing distances, as counted by individual single cells from 6 flow cells of the ONT platform. **d,** Percentage of reads with different library structures for barcodes at different edit distances, as counted by individual single cells from 6 flow cells of the ONT platform. HF and TR were the expected library structures. **e,** Percentage of reads that could be assigned to a single cell under consideration of barcode information and length of fragments. **f,** Number of template reads and non-template (complementary) reads in each cell, as counted by 96 individual single cells in one complete flow cell of the ONT platform. The dashed line indicated the average reads per single cell. **g,** Percentage of reads that passed the trimming procedure. **h,** Percentage of reads stratified by step-wise filter conditions, as counted by individual single cells from 6 flow cells of the ONT platform. **i,** Total read counts and duplication rate per cell (364 cells that passed QC) of NanoStrand-seq library.

Supplementary Fig. S3

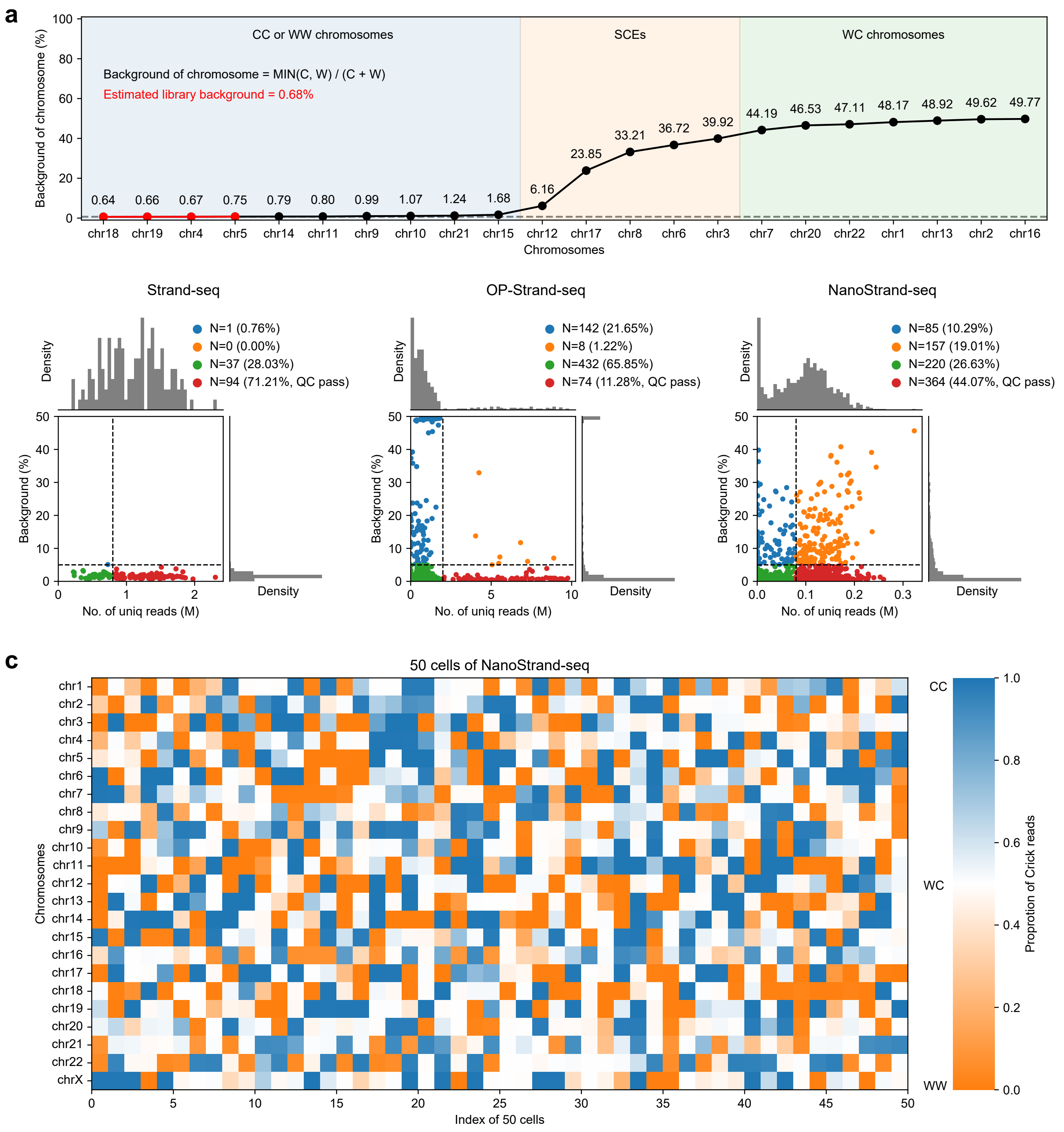

Supplementary information, Fig. S3 Estimation of background for NanoStrand-seq data.

a, Representative ideogram plot showing background estimation for a specific NanoStrand-seq library, corresponding to the single cell shown in Fig. 1g (see methods). Background near 50% indicated that the two homologs generated the opposite orientation of template strand reads (Watson-Crick or Crick-Watson), whereas background close to 0 indicated that two homologs generated the same orientation of template strand reads (Watson-Watson or Crick-Crick). Additionally, the intermediate background probably indicated SCEs. Note that when the SCEs located near the telomere, the background was also near 0 or 50%. The dashed line represented the average background per cell, counted by the mean value of four minimum background values of autosomes. **b,** Scatterplots showing the library quality of Strand-seq, OP-Strand-seq, and NanoStrand-seq. Each spot indicated a unique cell. The red spots represented cells that passed QC. **c,** Heatmap plot showing template strand inherited pattern of all chromosomes in 50 randomly selected cells that passed QC.

Supplementary Fig. S4

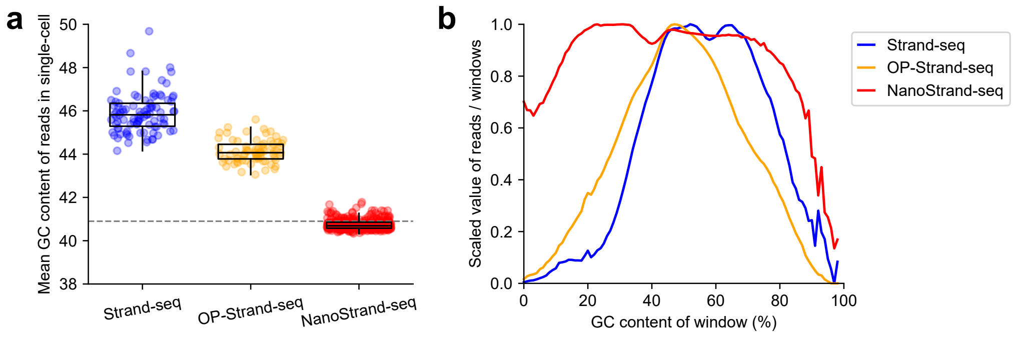

Supplementary information, Fig. S4 The GC content profile of NanoStrand-seq.
a, GC content of different approaches. The dashed line indicated the 40.9% human genomic GC content. **b,** The GC bias of different approaches.

Supplementary Fig. S5

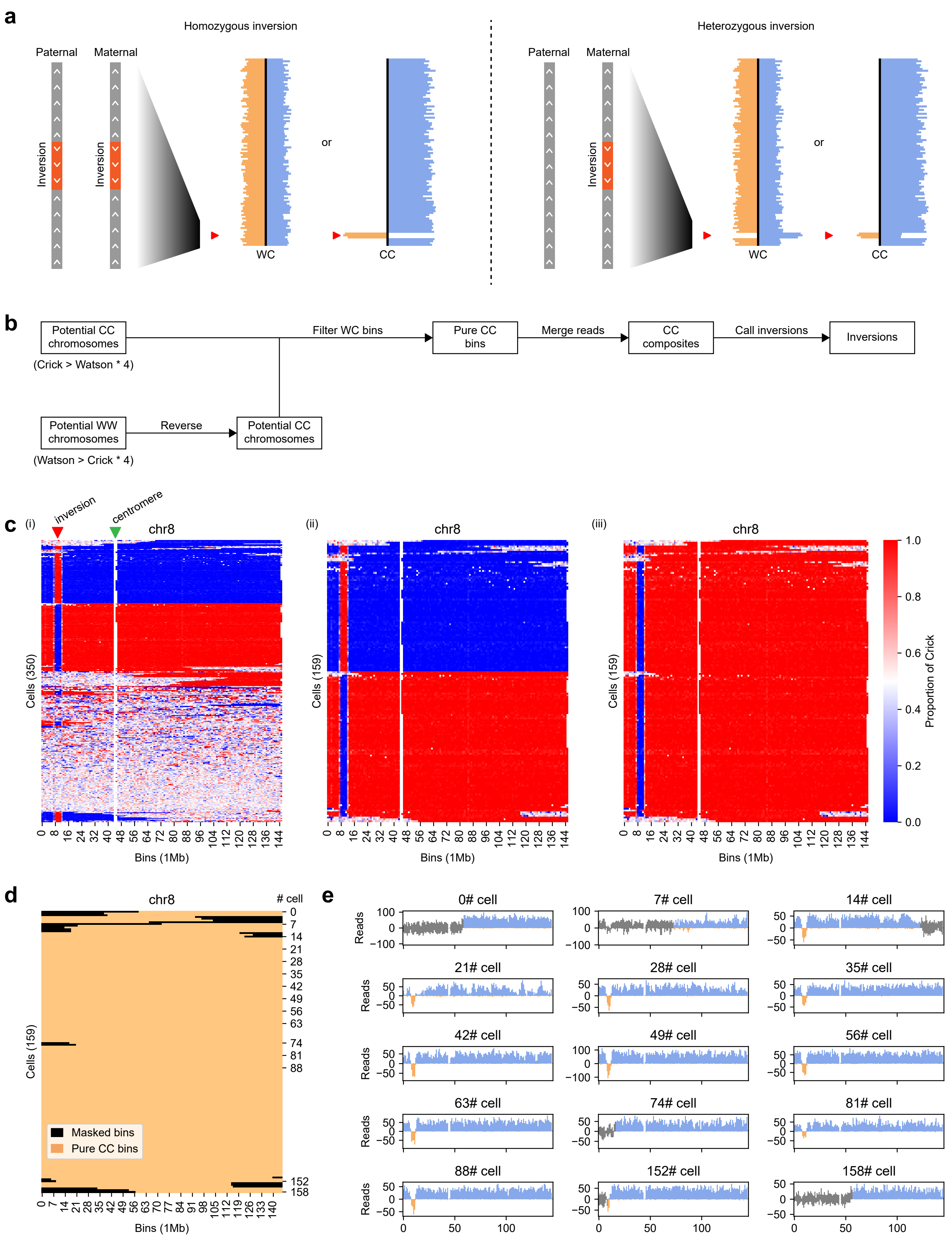

Supplementary information, Fig. S5 The strategy for detecting inversion events in NanoStrand-seq.

a, Schematic showing inversion patterns in NanoStrand-seq dataset. **b,** Pipeline for inversion identification. **c,** Process of cell clustering based on the template strand inherited state located on Chr8. Each row denoted a single cell (i). We then retained cells with only potential WW or CC state (ii) and switched WW tags of cells to CC tags (iii). In the end, inversions were visible as a recurrent change in strand state at the same position among all the cells. **d,** Histogram displaying genomic regions with continuous strand state (orange), while other regions were labeled as black. **e,** Examples of individual cells with visible inversion at the same position through pre-processing. Regions with WC patterns were masked as grey.

Supplementary Fig. S6

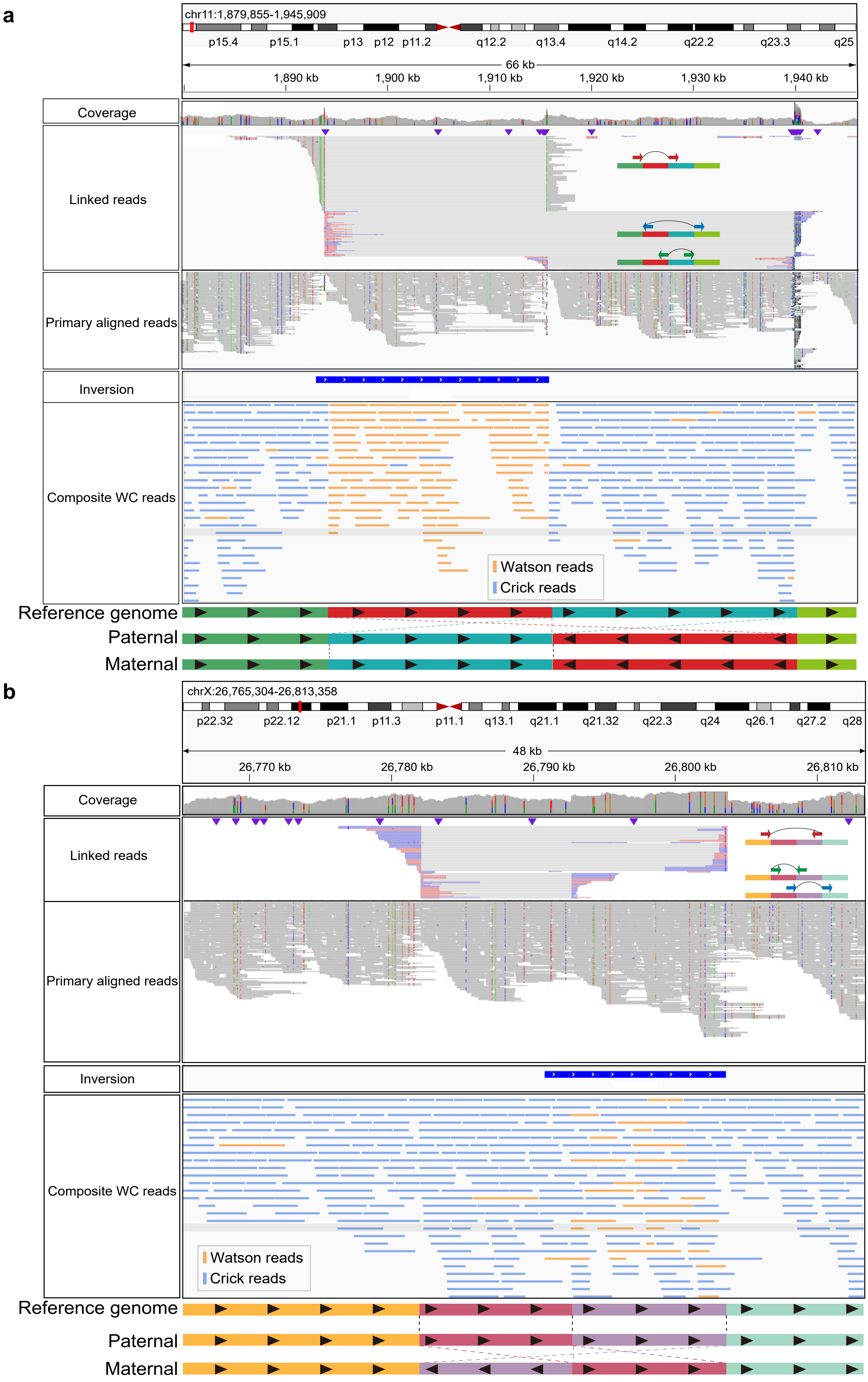

Supplementary information, Fig. S6 Examples of complex translocation events detected by NanoStrand-seq.

IGV showing the complex translocation in a haplotype-aware manner. **a,** Both haplotypes occurred a translocation event relative to the reference genome, and the translocated element was inverted, with the strand directionality opposite to the reference genome. **b,** Haplotype 1 was the same as the reference genome, while haplotype 2 occurred translocation, and was inverted during translocation. The parental information was annotated by GIAB.

Supplementary Fig. S7

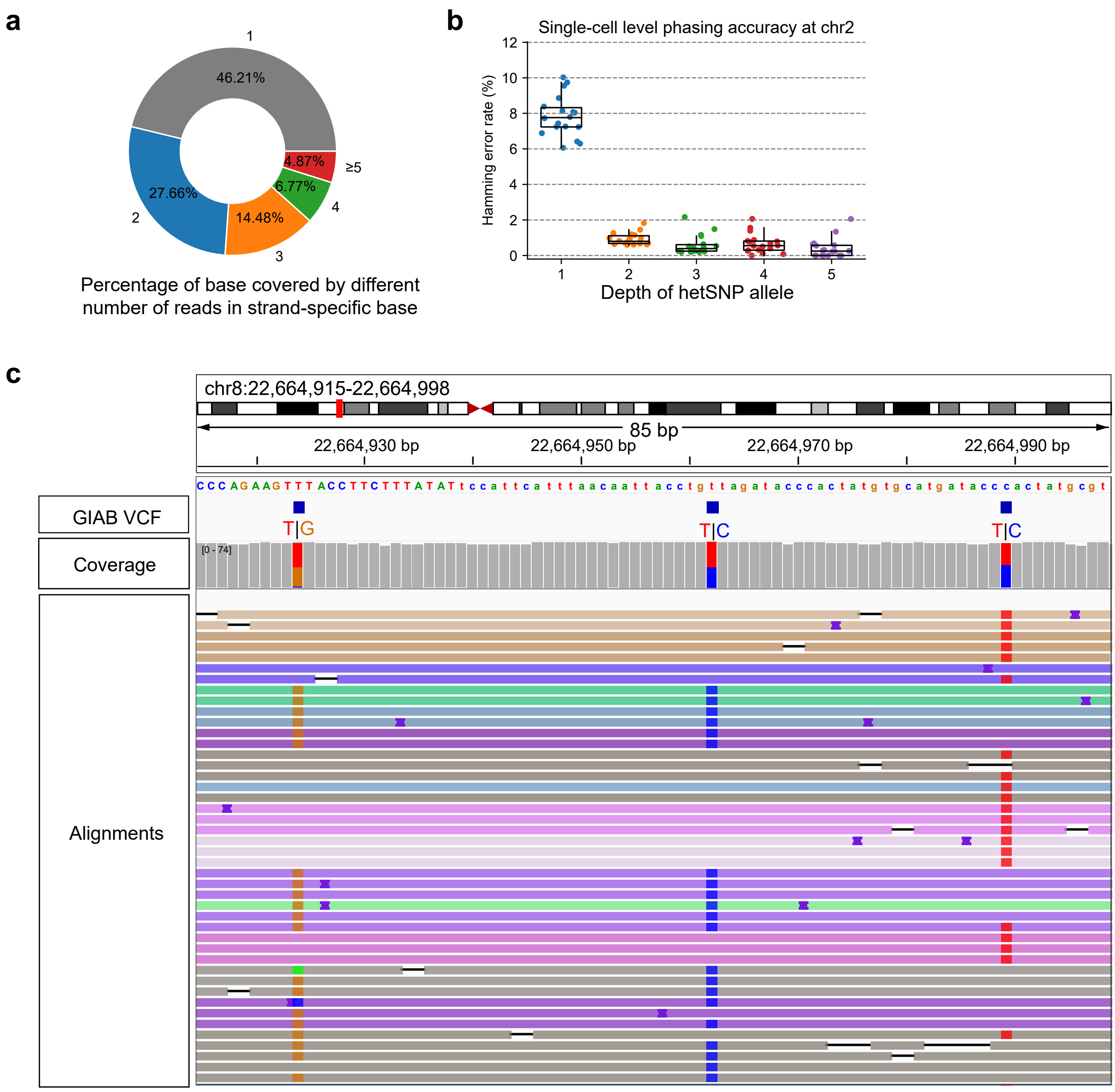

Supplementary information, Fig. S7 Representative performance of phasing by using NanoStrand-seq reads.

a, Pie chart showing the percentage of the base covered by different numbers of reads within strand-specific bases in individual cells (excluded zero-depth). For example, the pie chart showed that among all the covered base loci of each haplotype from individual cells, 46.21% of these base sites were covered only once. **b,** Representative example of the Hamming error rate at the different depths of heterozygous allele on Chr2 for randomly selected 18 individual cells with the WC pattern. **c,** Representative example of hetSNPs phasing using NanoStrand-seq reads. Each color denoted reads derived from the same cell.

Supplementary Fig. S8

a

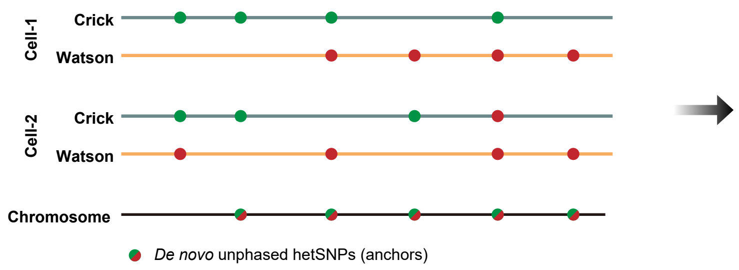

Pairwise comparison matrix

Cell-1	Cell-2	Overlap	Same	Different
Crick	Crick	3	2	1
Crick	Watson	3	0	3
Watson	Crick	2	1	1
Watson	Watson	3	3	0

N1 = 2 + 3 + 1 + 3 = 9

N2 = 1 + 0 + 1 +0 = 2

$\log_2$(Fold Change) = $\log_2$(N1 / N2) = 2.17 > 0

b

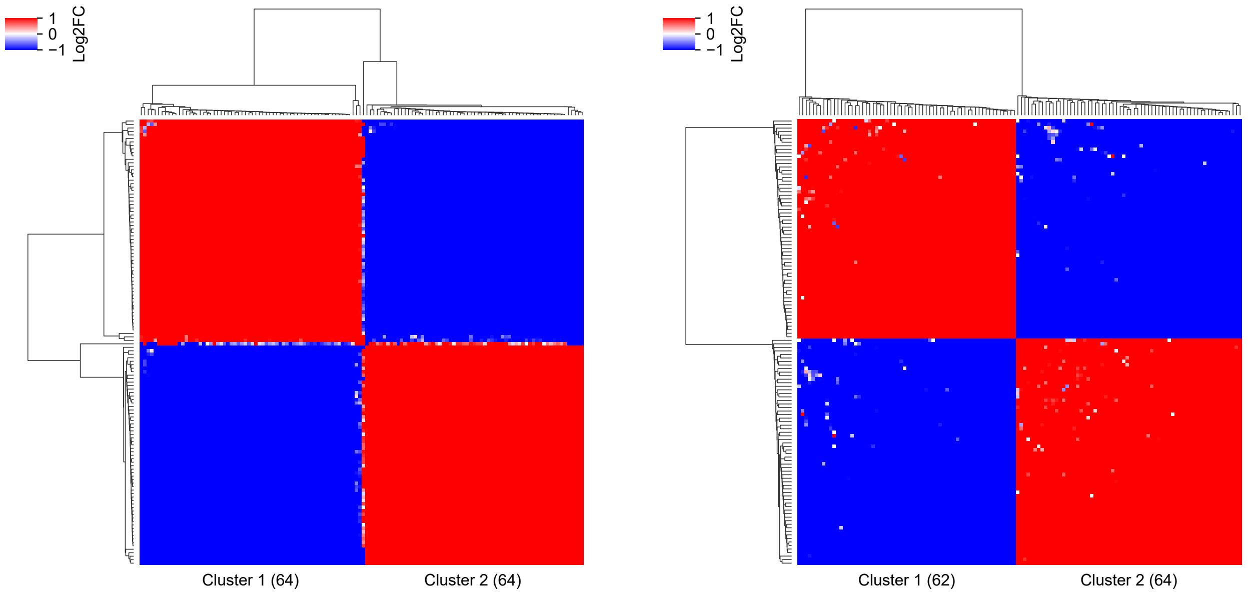

Chromosome 1

Chromosome 22

Supplementary information, Fig. S8 The strategy for clustering cells based on *de novo* called hetSNPs.

a, A depiction of the similarity of template strand inherited patterns in each chromosome between paired cells with WC pattern, by querying the specific base both on the Crick template strands and Watson template strands at positions given by hetSNPs (called by pseudo-bulk NanoStrand-seq) and calculating the similarity between paired cells. The similarity was calculated as Log_2(Fold Change). **b,** Cell clustering of the longest Chr1 and the shortest Chr22. Log_2(Fold Change) larger than 1 was shown as 1, indicating that reads in the same template strands of two paired cells were from one identical haplotype, while Log_2(Fold Change) smaller than -1 was shown as -1, indicating that the reads in the same template strands of two paired cells were from different haplotypes. White dots indicated that there were no overlapping hetSNPs between the corresponding paired cells or failed to judge.

Supplementary Fig. S9

a

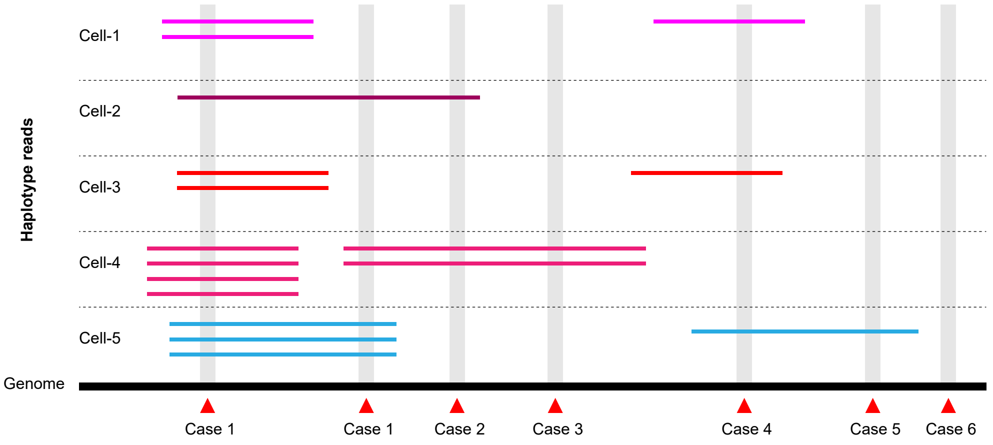

b

Case	No. of cells with 1 read (C1)	No. of cells with ≥ 2 reads (C2)	Condition of ALLELE consistent
1	0,1,2,3,4,...	2,3,4,...	Ignore C1. For C2, percentage of reads in one cell that support ALLELE ≥ 75%, and percentage of C2 that support ALLELE ≥ 75%.
2	1,2,3,4,...	1	For C1, C1 ≥ 2, percentage of C1 supporting ALLELE ≥ 75%. For C2, percentage of reads supporting ALLELE ≥ 75%.
3	0	1	For C2, reads ≥ 4 and percentage of reads supporting ALLELE ≥ 75%.
4	2,3,4,...	0	For C1, percentage of C1 supporting ALLELE ≥ 75%.
5	1	0	Always consistent.
6	0	0	Empty.

c

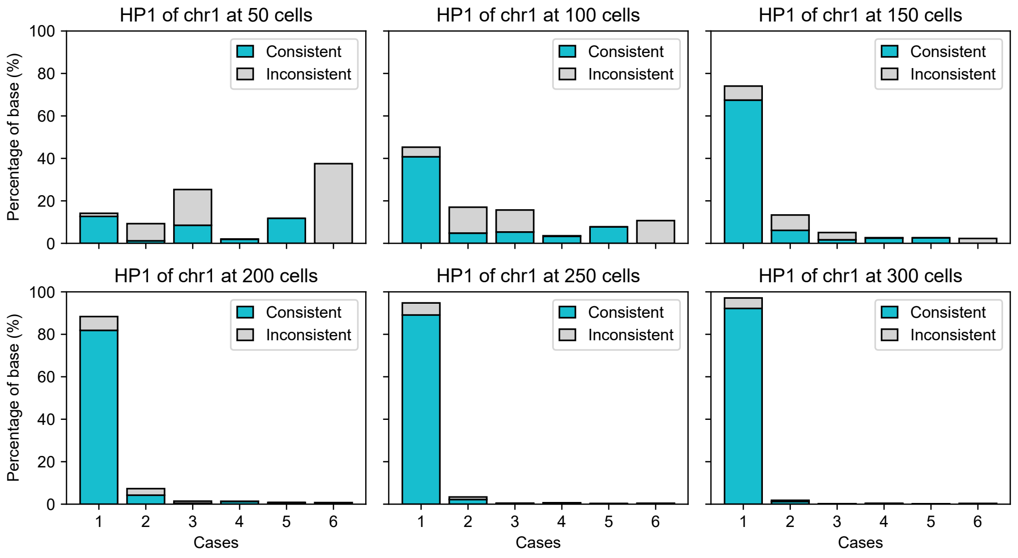

Supplementary information, Fig. S9 Overview of haplotype-resolved SNP calling strategy.

a, Schematic showing possible cases covered by haplotype-resolved reads at each position across the whole genome. **b,** Categorization of SNP calling across the whole genome at different supporting evidence thresholds (based on reads per cell and corresponding cell numbers) using haplotype-partitioned reads. Nucleotides that successfully passed the filtering criteria were denoted as 'consistent', otherwise, they would be denoted as 'inconsistent'. In detail, in case 1, a position was covered by more than 1 cell, and each cell contained more than 1 read. We ignored the cells with only 1 read. For a cell, ≥ 75% of reads supported a certain base, and ≥ 75% of cells supported an identical base, then the base was denoted as 'consistent'. In case 2, a position was covered by 1 cell that contained more than 1 read, and simultaneously covered by multiple cells that contained only 1 read. For cells with more than 1 read, ≥ 75% of reads supported a certain base, and for cells with only 1 read, ≥ 75% of cells supported an identical base, then the base was denoted as 'consistent'. In case 3, a position was covered by 1 cell that contained more than 1 read. In this cell, ≥ 75% of reads supported a certain base and then the base was denoted as 'consistent'. In case 4, a position was covered by more than 1 cell that contained only 1 read. The base was denoted as 'consistent' if ≥ 75% of cells supported an identical base. In case 5, a position was covered by 1 cell that contained only 1 read. The base was always denoted as 'consistent'. In case 6, a position without any supporting read was considered empty. Note that we ultimately designated consistent nucleotides from cases 1-3 to generate the genome-wide high-confidence calls for both haplotypes. **c,** Histogram showing the proportion of consistent and inconsistent nucleotides in each case using haplotyped reads located on the high-confidence regions of Chr1. The high-confidence regions were annotated by GIAB.

Supplementary Fig. S10

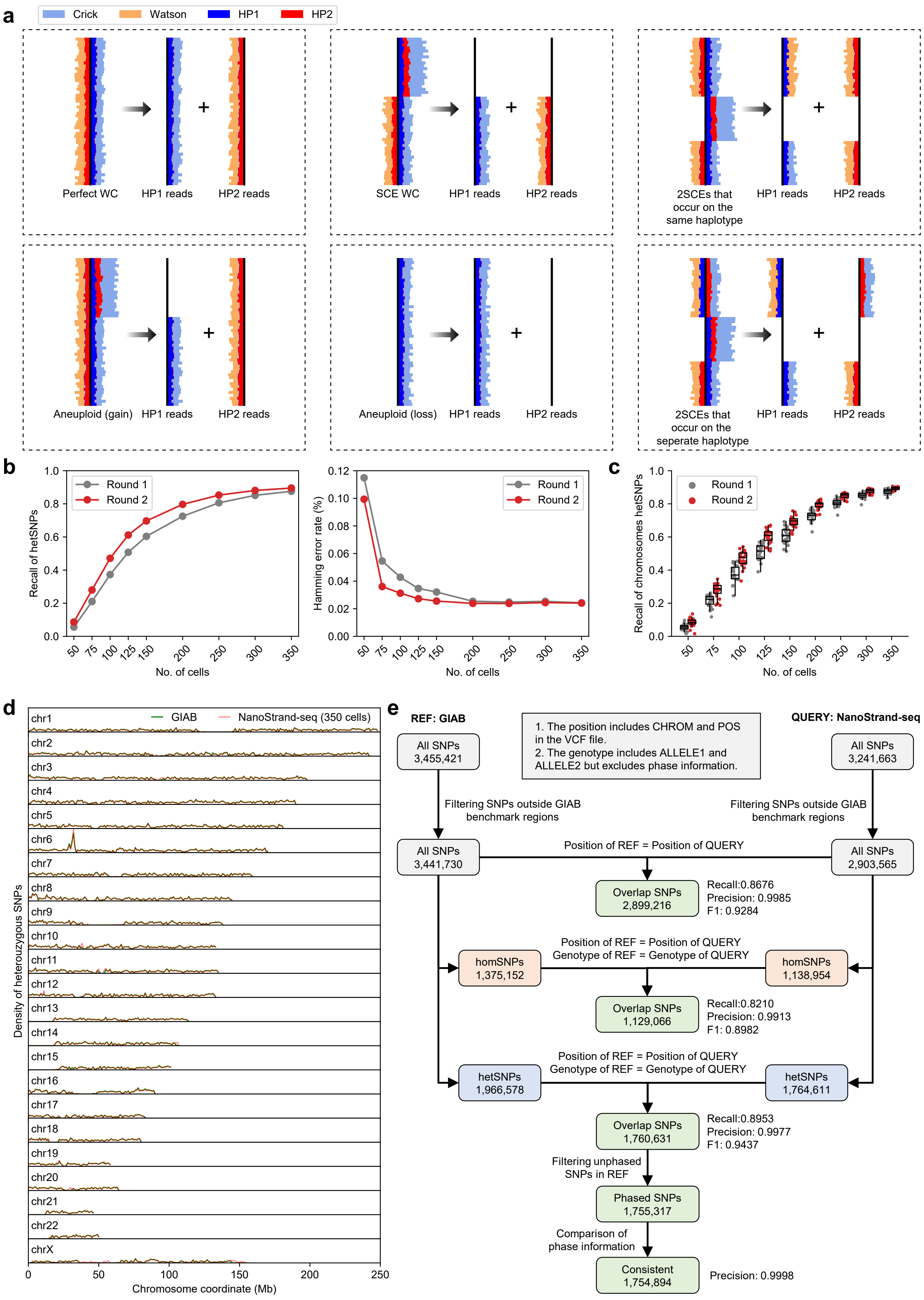

Supplementary information, Fig. S10 Performance of round 2 haplotype reconstruction.

a, Illustrations of tagging each cell by phase information based on round 1 haplotype reconstruction-derived hetSNPs and extracting the reads in the regions that were only assigned to one haplotype. **b,** Comparison of performance between round 1 reconstruction and round 2 reconstruction. **c,** Comparison of recall rate of each chromosome at different supporting cell numbers. **d,** The distribution of hetSNPs on each chromosome. The light red line (NanoStrand-seq) was highly consistent with the light green line (GIAB), indicating that NanoStrand-seq was a powerful technique for detecting SNPs. **e,** Flowchat of benchmark process of SNPs derived from NanoStrand-seq data.

Supplementary Fig. S11

a

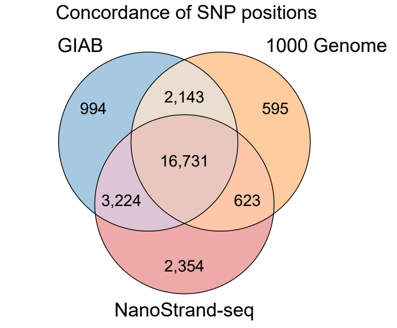

b

Genotyping accuracy at MHC region

GIAB	1000Genome	NanoStrand-seq	Number
0/1	0/1	0/1	11,665
1/1	1/1	1/1	4,857
0/1	1/1	0/1	115
0/1	0/1	1/1	43
1/1	0/1	1/1	17
1/2	2/2	0/2	15
1/2	2/2	2/2	8
1/1	1/1	0/1	5
1/2	2/2	1/2	1
1/2	0/2	0/2	1
1/2	2/2	2/3	1
1/2	1/1	1/2	1
1/2	0/2	2/2	1
1/3	3/3	2/3	1

NanoStrand-seq vs. GIAB:
Genotyping accuracy = 99.55%

NanoStrand-seq vs. 1000Genome:
Genotyping accuracy = 98.80%

c

Phasing accuracy at MHC region

GIAB	1000Genome	NanoStrand-seq	Number
0|1	0|1	0|1	6,082
1|0	1|0	1|0	5,455
0|1	1|0	0|1	38
1|0	0|1	1|0	87

NanoStrand-seq vs. GIAB:
Hamming error rate = 0%

NanoStrand-seq vs. 1000Genome:
Hamming error rate = 1.07%

d

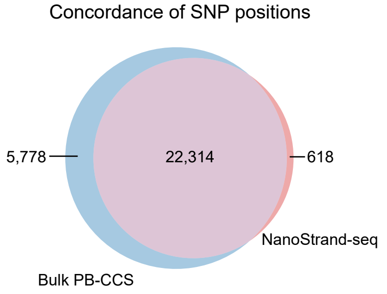

e

Genotyping accuracy at MHC region

Bulk PB-CCS	NanoStrand-seq	Number
HOM	HOM	5,934
HOM	HOM.2	5
HOM	HET	79
HET	HOM	131
HET	HET	16,156
HET	HET.2	9

NanoStrand-seq vs. Bulk PB-CCS:
Genotyping accuracy = 99.00%

f

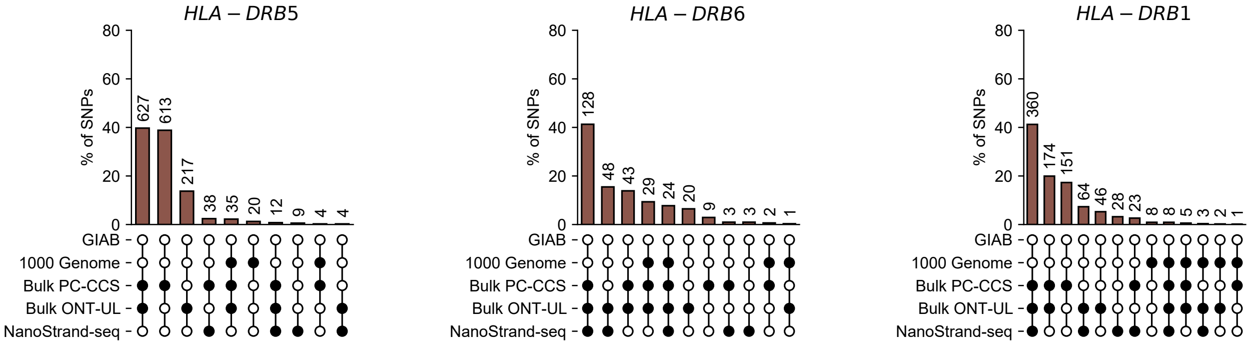

Supplementary information, Fig. S11 Comparison of SNPs detected in MHC region from different call sets.

a, Venn diagram depicting the overlap of SNP positions in the MHC region among NanoStrand-seq, GIAB, and 1000 Genome. **b,** The genotyping performance of NanoStrand-seq in the MHC region relative to GIAB and 1000 Genome. **c,** The phasing performance of NanoStrand-seq in the MHC region relative to GIAB and 1000 Genome. **d,** Venn diagram depicting the overlap of SNP positions in MHC region between NanoStrand-seq and bulk PB-CCS data. **e,** The genotyping consistency between NanoStrand-seq and bulk PB-CCS data in MHC region. **f,** The concordance of SNPs in three *HLA* genes (*HLA-DRB5*, *HLA-DRB6*, and *HLA-DRB1*) in five different call sets.

Supplementary Fig. S12

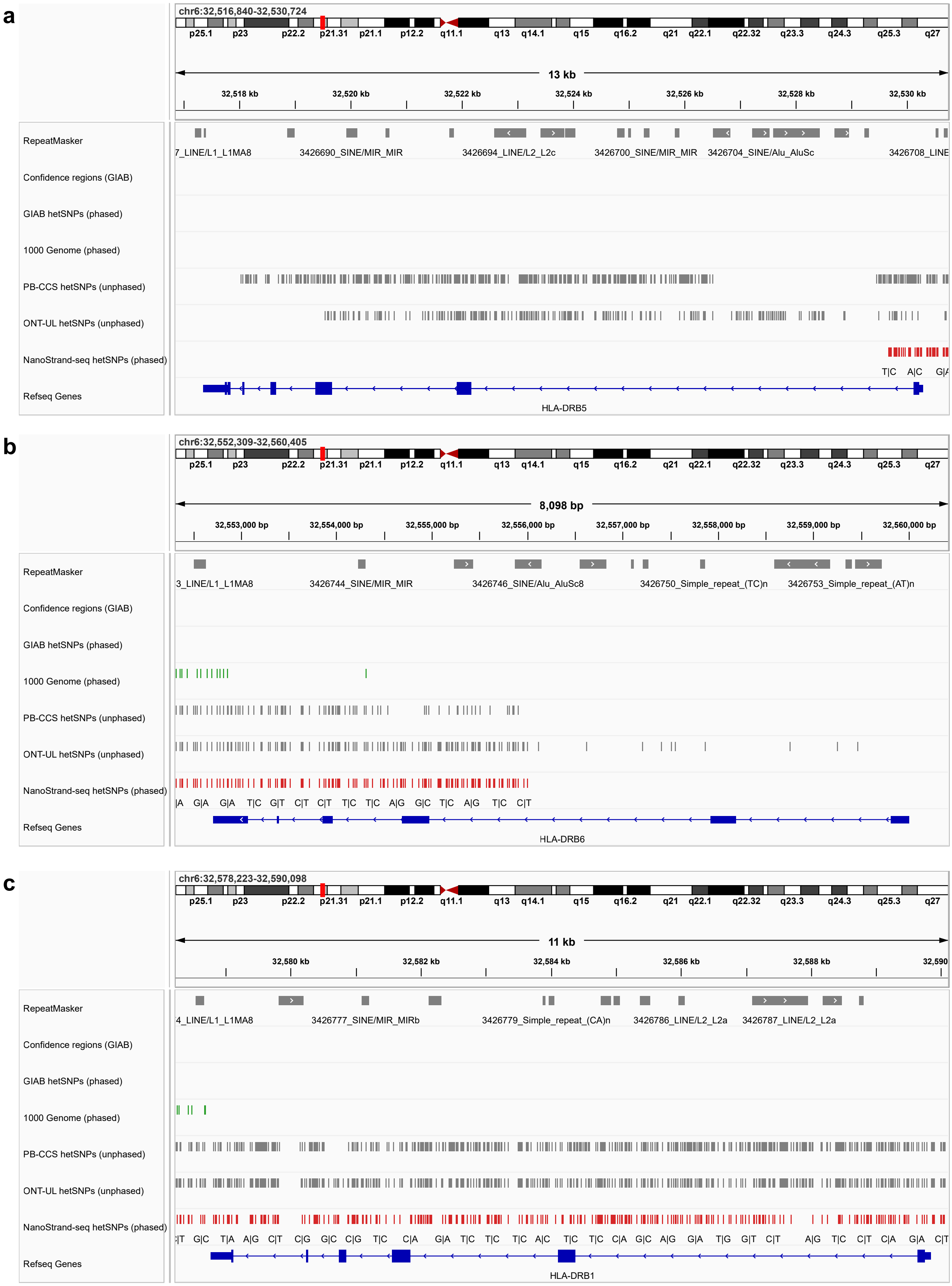

Supplementary information, Fig. S12 Additional SNPs were detected using NanoStrand-seq compared to GIAB.

a, b, c, IGV showing that distribution of SNP positions detected by five different call sets on three *HLA* genes (*HLA-DRB5*, *HLA-DRB6*, and *HLA-DRB1*).

Supplementary Fig. S13

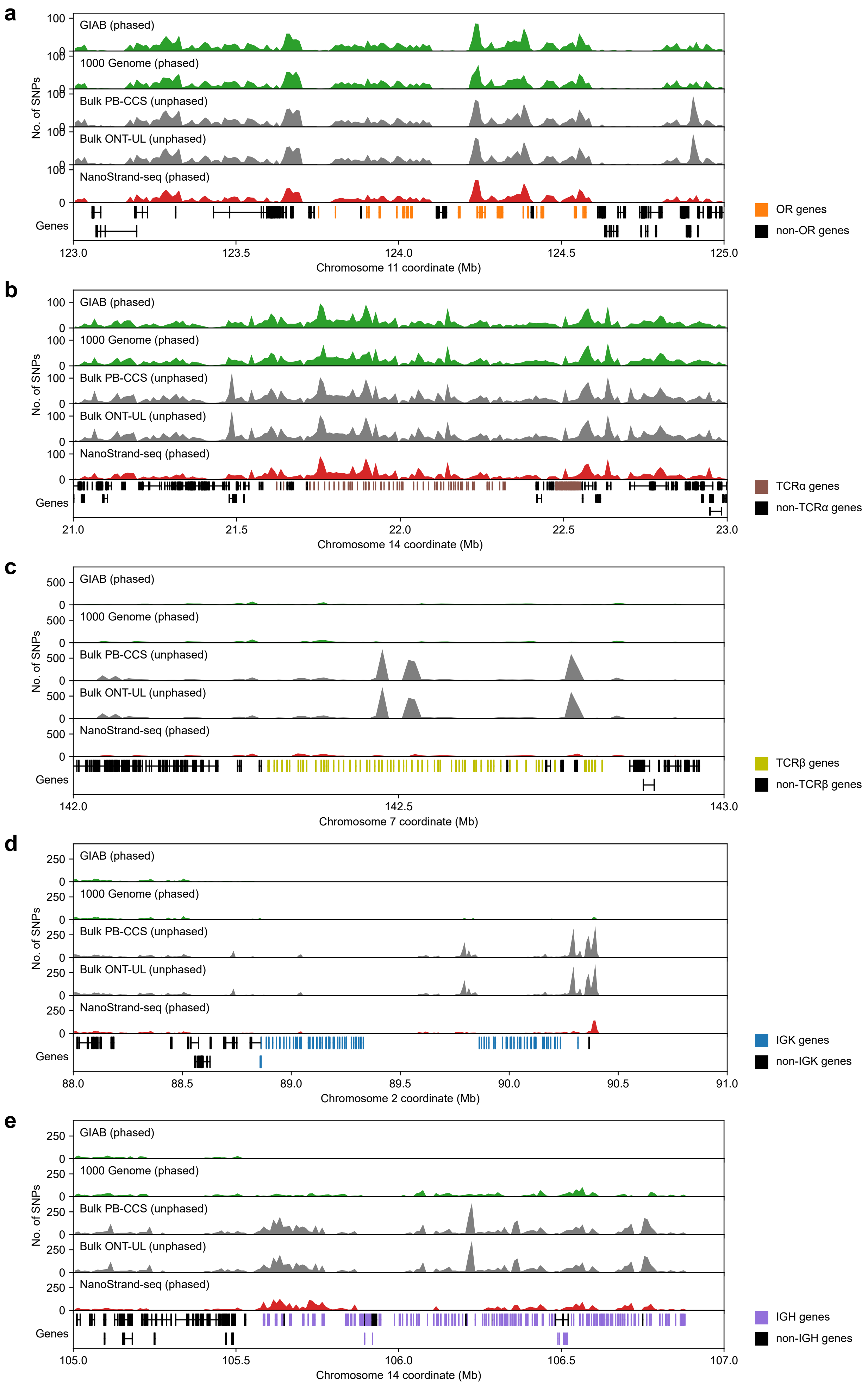

Supplementary information, Fig. S13 The consistency of SNP calling among different approaches.

Representative examples of SNP calling among five different call sets at *OR* gene cluster (**a**), *TCRα* genes (**b**), and *TCRβ* genes (**c**), *IGK* genes (**d**), and *IGH* genes (**e**).

Supplementary Fig. S14

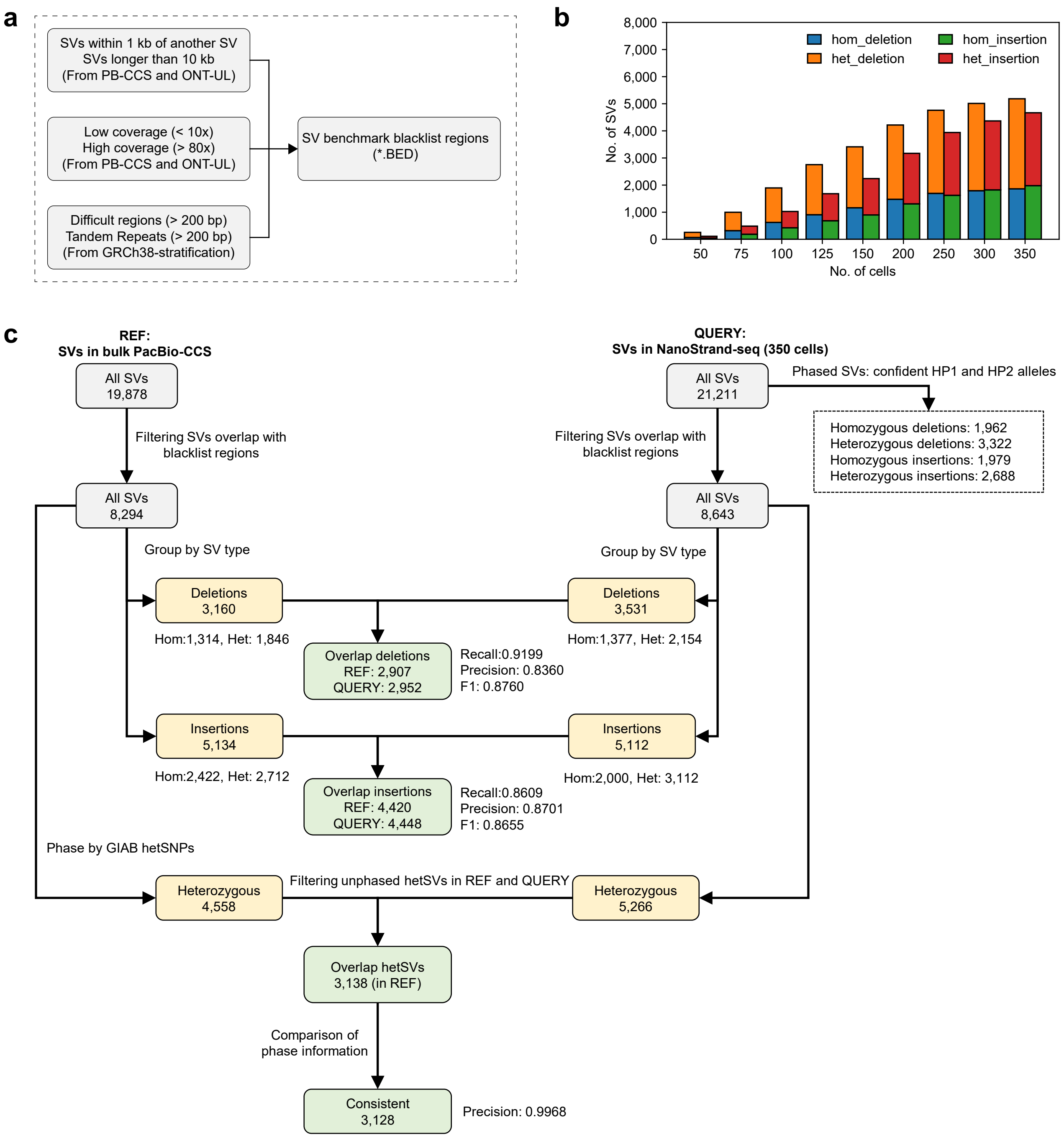

Supplementary information, Fig. S14 The blacklist regions and number of SVs detected by NanoStrand-seq.

a, The criteria for SV benchmark blacklist regions. **b,** Histogram showing the counts of SVs (homozygous SVs and phased heterozygous SVs) detected at given cell numbers. **c,** Flowchat of the benchmark process of SVs derived from NanoStrand-seq data.

Supplementary Fig. S15

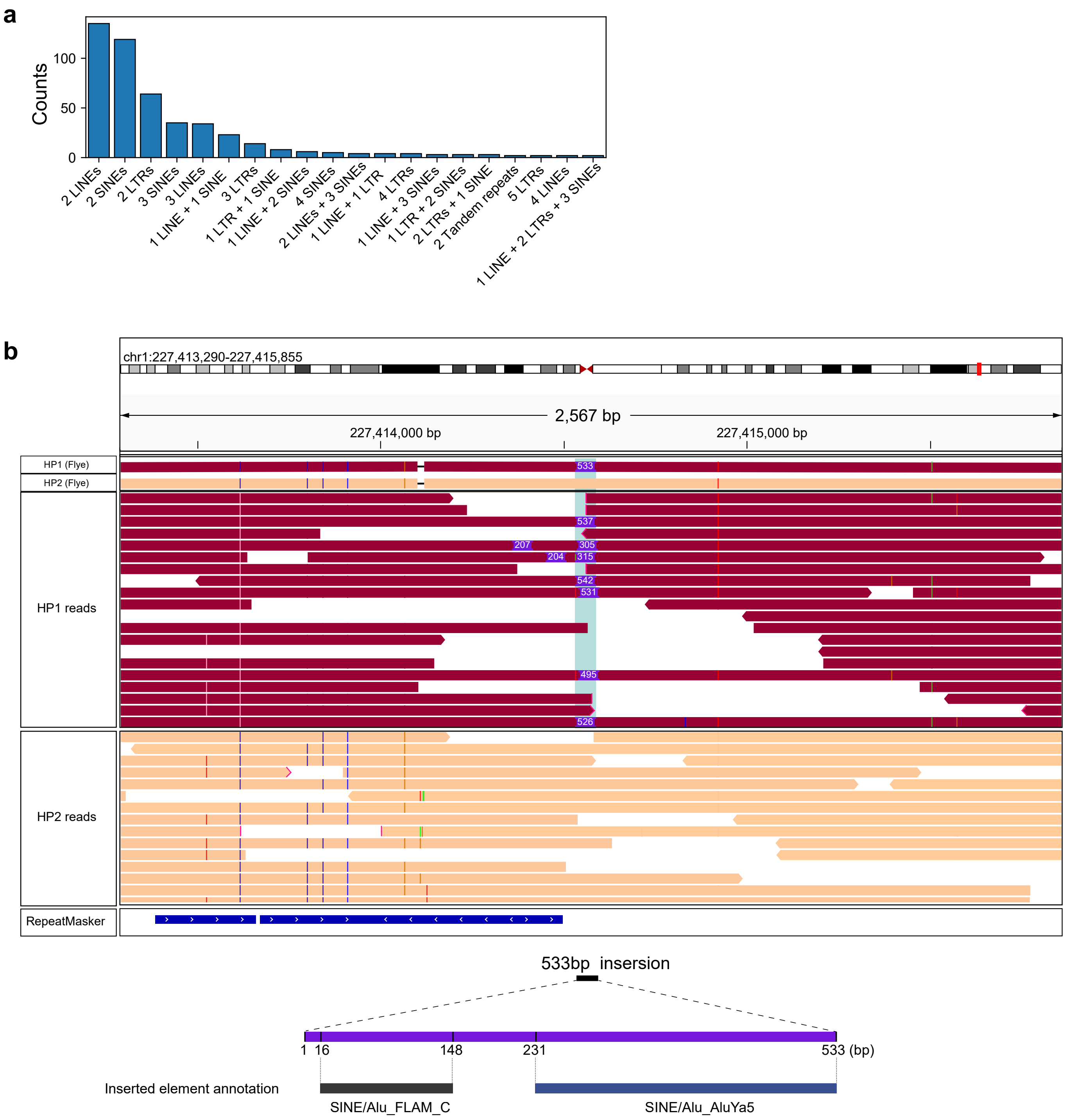

Supplementary information, Fig. S15 Examples of multiple repetitive elements within a single SV event detected using NanoStrand-seq.

a, Number of repetitive element combinations in phased SV call sets in NanoStrand-seq data. **b,** IGV showing that NanoStrand-seq could efficiently phase SVs that consist of two distinct SINE/Alu subtypes.

Supplementary Fig. S16

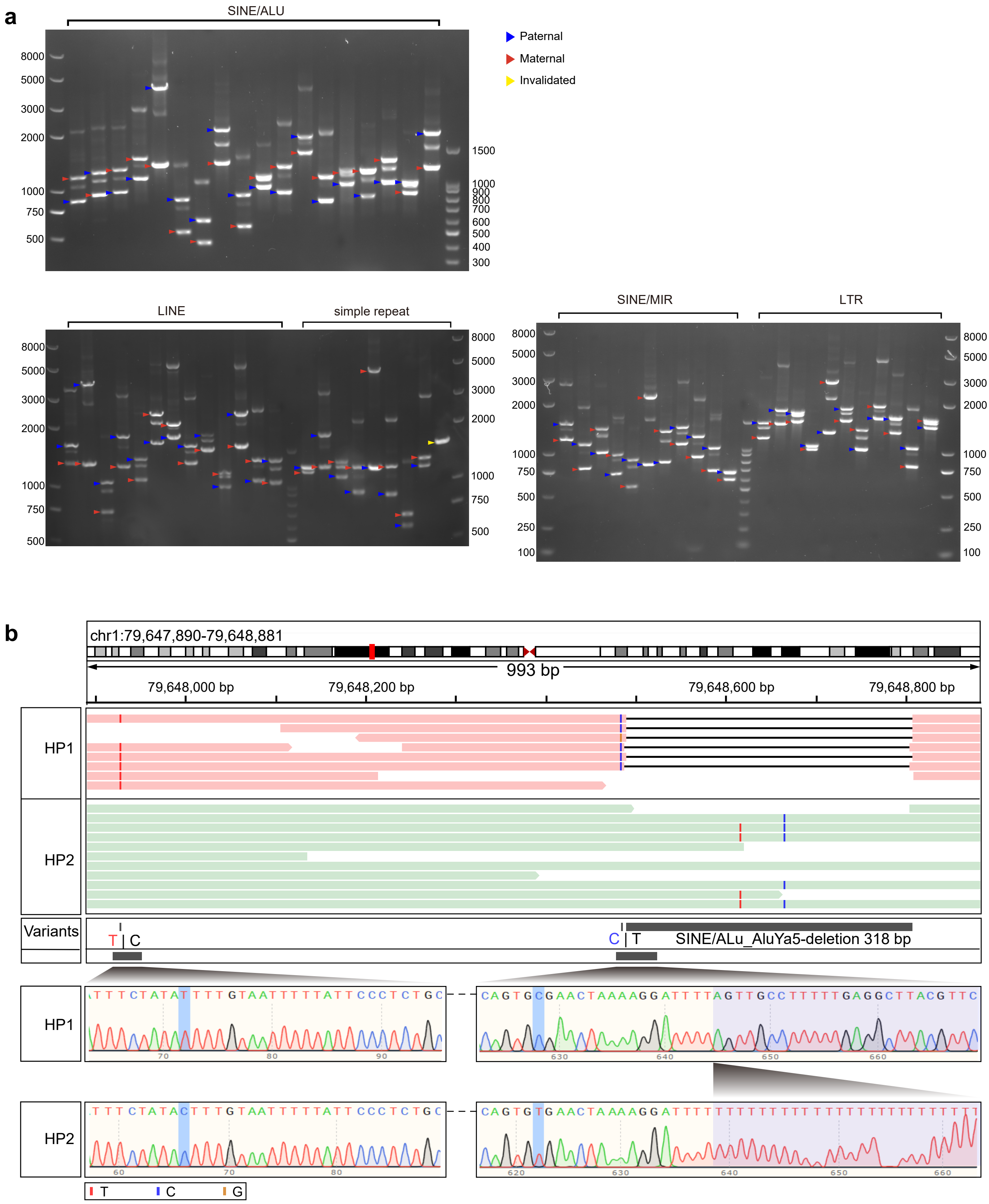

Supplementary information, Fig. S16 Validation of haplotype-specific SVs identified by NanoStrand-seq.

a, PCR analysis of genomic DNA to validate haplotype-specific SV events including SINE/Alu, LINE, simple repeat, SINE/MIR, and LTR. Blue triangles indicated the paternal allele, while red triangles indicated the maternal allele. The parental origin of hetSNPs was annotated by GIAB. We collected both paternal and maternal bands, applied them to Sanger sequencing, and validated the corresponding linkage of hetSNPs and SV breakpoints. The DNA size marker was shown in base pairs. **b,** Representative example of haplotype-specific SVs (a deletion event belonging to SINE/Alu repetitive elements) validated by Sanger sequencing. Blue and purple areas in Sanger sequencing results highlighted hetSNPs and SV edge, respectively.

Supplementary Fig. S17

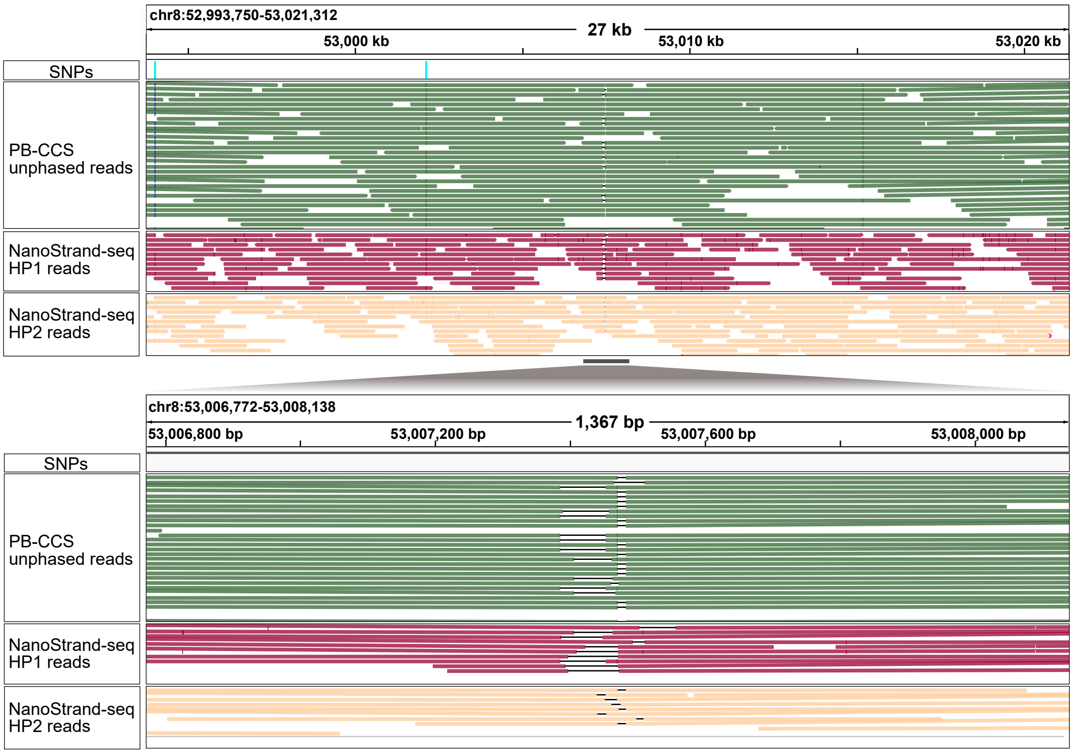

Supplementary information, Fig. S17 The SV phasing performance of NanoStrand-seq in hetSNP-poor regions.

IGV showing that NanoStrand-seq could directly phase SV which was approximately 14 kb away from its nearest hetSNP, while PB-CCS reads could not phase. The bottom panel showed the zoomed-in window of this SV. We showed a 67 bp deletion on the paternal allele (HP1), and a 12 bp indel on the maternal allele (HP2) compared to the reference genome. The haplotype origin was annotated by GIAB.

Supplementary Fig. S18

a

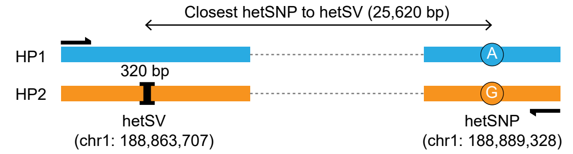

Supporting reads ratio = 83.00%

Combination (SNP-SV)	Supporting reads		
	Bulk PB-CCS (GIAB)	Bulk ONT-UL (GIAB)	Targeted ONT
A-REF	0	0	96
A-INS	0	0	17
G-REF	0	0	17
G-INS	0	0	70
Others	0	0	0

b

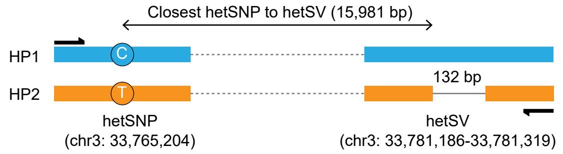

Supporting reads ratio = 84.50%

Combination (SNP-SV)	Supporting reads		
	Bulk PB-CCS (GIAB)	Bulk ONT-UL (GIAB)	Targeted ONT
C-REF	0	0	88
C-DEL	0	0	16
T-REF	0	0	15
T-DEL	0	1	81
Others	0	0	0

c

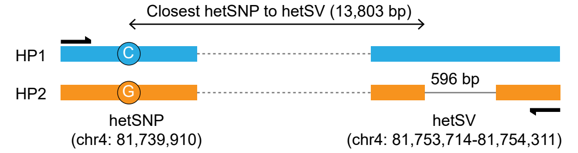

Supporting reads ratio = 87.00%

Combination (SNP-SV)	Supporting reads		
	Bulk PB-CCS (GIAB)	Bulk ONT-UL (GIAB)	Targeted ONT
C-REF	0	1	82
C-DEL	0	0	10
G-REF	0	0	16
G-DEL	0	0	92
Others	0	0	0

d

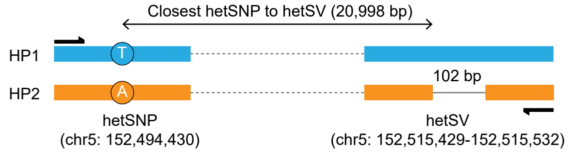

Supporting reads ratio = 81.00%

Combination (SNP-SV)	Supporting reads		
	Bulk PB-CCS (GIAB)	Bulk ONT-UL (GIAB)	Targeted ONT
T-REF	0	0	70
T-DEL	0	0	22
A-REF	0	0	16
A-DEL	0	0	92
Others	0	0	0

e

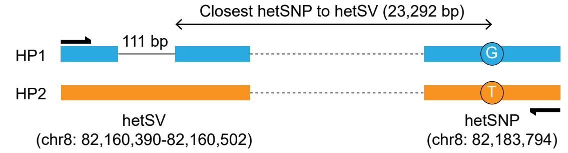

Supporting reads ratio = 86.00%

Combination (SNP-SV)	Supporting reads		
	Bulk PB-CCS (GIAB)	Bulk ONT-UL (GIAB)	Targeted ONT
G-REF	0	0	14
G-DEL	0	0	86
T-REF	0	0	86
T-DEL	0	0	14
Others	0	0	0

f

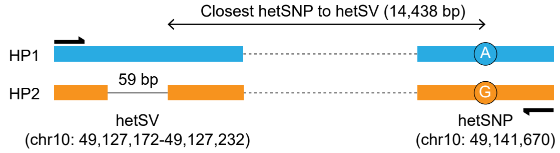

Supporting reads ratio = 85.00%

Combination (SNP-SV)	Supporting reads		
	Bulk PB-CCS (GIAB)	Bulk ONT-UL (GIAB)	Targeted ONT
A-REF	0	0	91
A-DEL	0	0	13
G-REF	0	0	17
G-DEL	0	1	79
Others	0	0	0

g

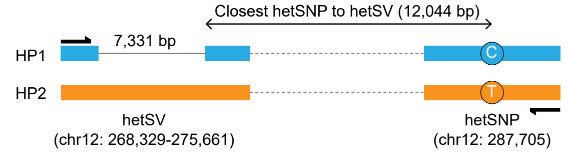

Supporting reads ratio = 92.00%

Combination (SNP-SV)	Supporting reads		
	Bulk PB-CCS (GIAB)	Bulk ONT-UL (GIAB)	Targeted ONT
C-REF	0	0	3
C-DEL	0	0	173
T-REF	0	0	11
T-DEL	0	0	13
Others	0	0	0

h

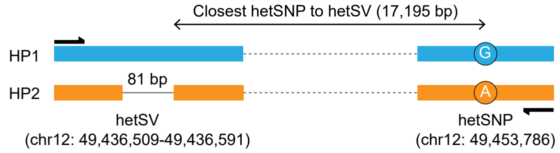

Supporting reads ratio = 79.50%

Combination (SNP-SV)	Supporting reads		
	Bulk PB-CCS (GIAB)	Bulk ONT-UL (GIAB)	Targeted ONT
G-REF	0	0	84
G-DEL	0	0	14
A-REF	0	0	27
A-DEL	0	0	75
Others	0	0	0

i

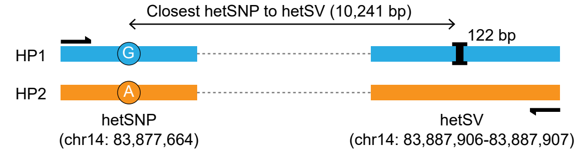

Supporting reads ratio = 85.50%

Combination (SNP-SV)	Supporting reads		
	Bulk PB-CCS (GIAB)	Bulk ONT-UL (GIAB)	Targeted ONT
G-REF	0	0	13
G-INS	0	2	81
A-REF	0	1	90
A-INS	0	0	15
Others	0	0	1

Supplementary information, Fig. S18 Validation of hetSVs that were more than 10 kb away from their nearest hetSNPs.

a-i, We validated the hetSVs which were more than 10 kb away from their nearest hetSNPs. The genetic linkage of these hetSVs and their nearest hetSNPs was detected by NanoStrand-seq. To validate these hetSVs, we performed PCR and subjected the PCR products to Nanopore sequencing (targeted ONT). Black arrows indicated PCR primer binding sites (100-5000 bp away from the region detected). The tables showed the number of reads supporting the linkage information of genetic variants from bulk PB-CCS, bulk ONT-UL, and target ONT. As shown in the tables, in all these 9 cases, on average 85% (79% - 92%) of amplicons supported the phasing information defined by NanoStrand-seq, while bulk PB-CCS and bulk ONT-UL data failed. Note that a small number of amplicons conflicted with the prediction, which may be due to the incorrect amplification by primers from the truncated PCR products.

Supplementary Fig. S19

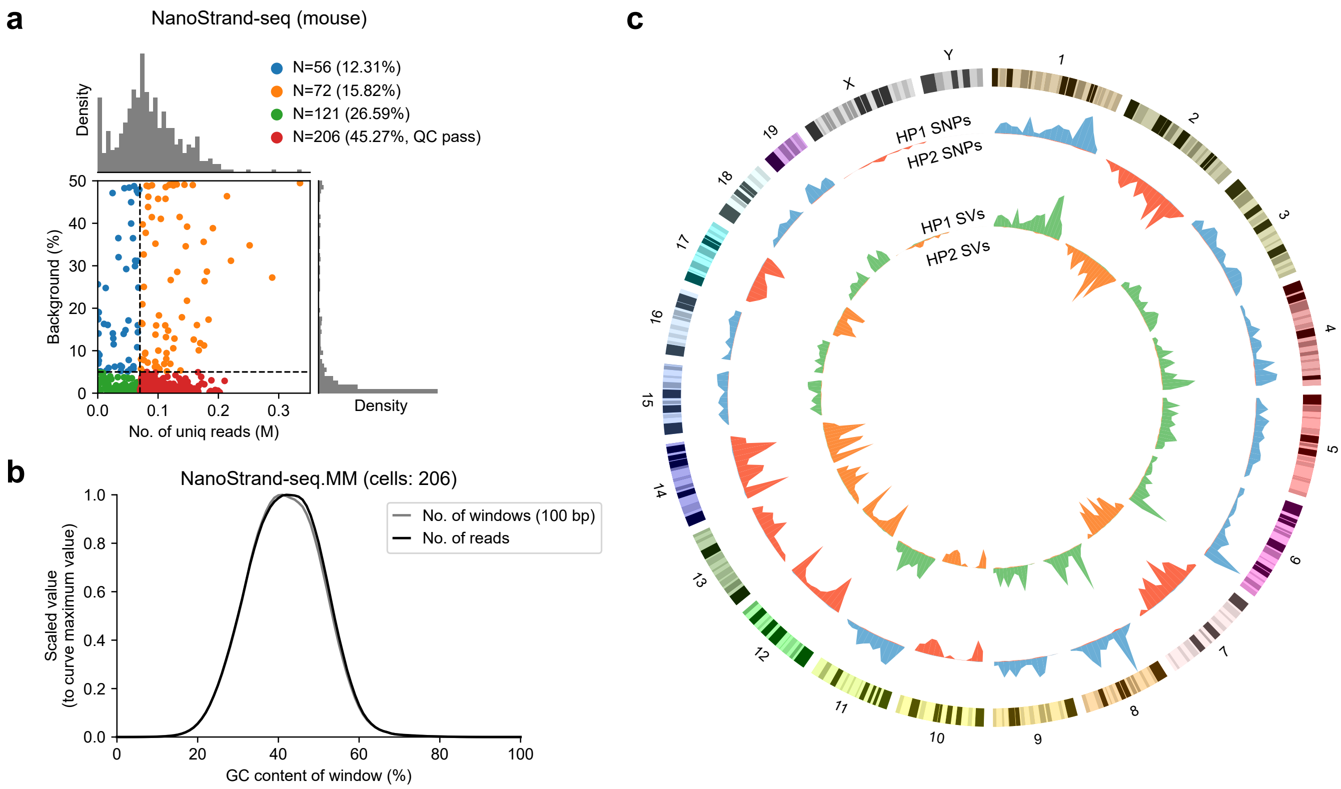

Supplementary information, Fig. S19 Characterization of NanoStrand-seq data in mouse primary cultured cells.

a, Scatterplot showing library quality based on background and unique read number of primary mouse cells. **b,** Plots of GC bias of NanoStrand-seq in primary mouse cells. The grey lines indicated the relative quantity within different GC content windows in the genome, and the black lines showed the relative quantity of reads within different GC content windows obtained by NanoStrand-seq. **c,** Circos plot illustrating the distribution of SNPs (middle) and SVs (inner) in the whole genome (outer) for each haplotype. Bin size = 10 Mb.

Supplementary Fig. S20

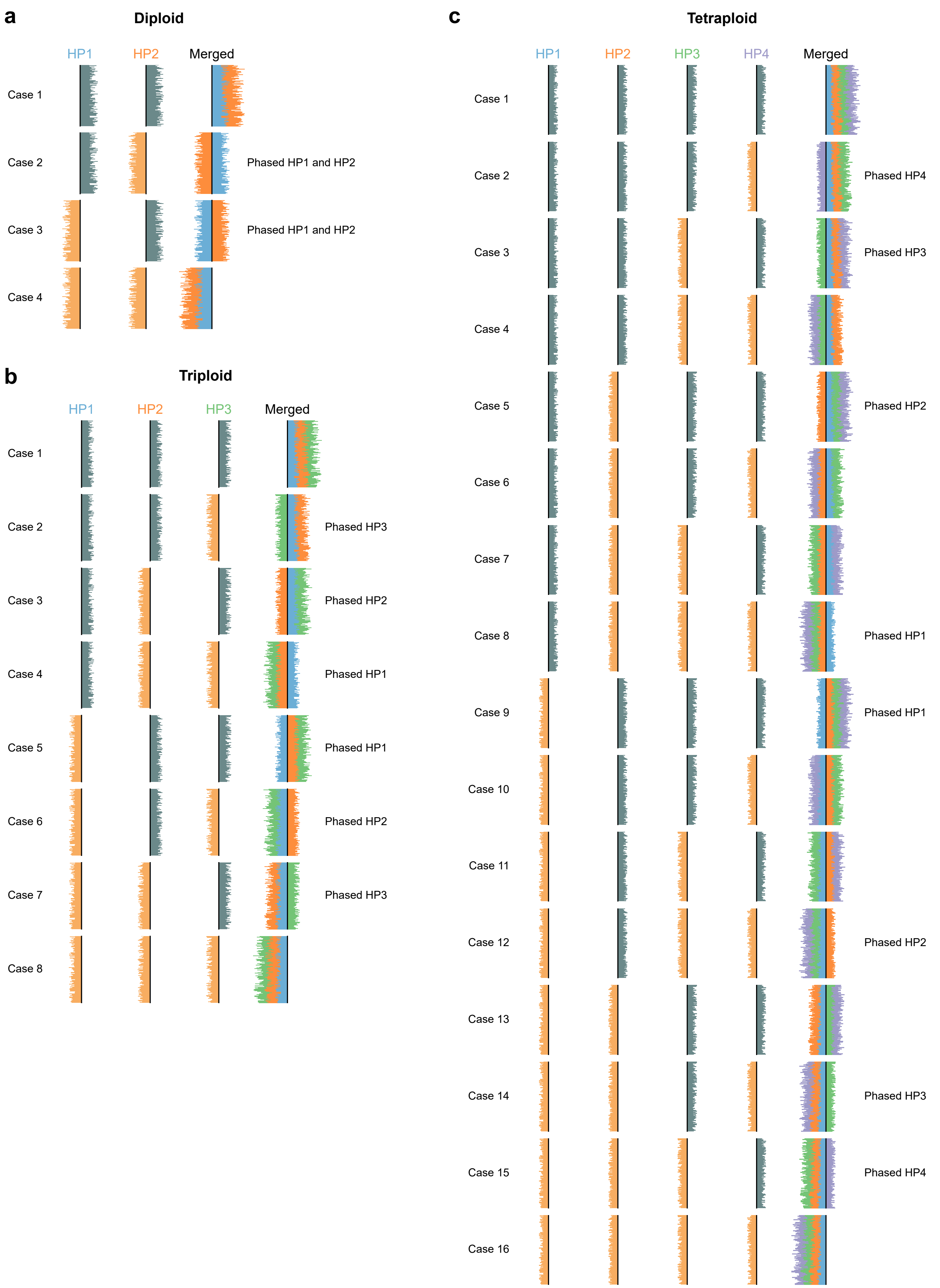

Supplementary information, Fig. S20 Schematic diagram of haplotype phasing using NanoStrand-seq in polyploid genomes.

a, In the diploid genome, HP1 and HP2 can be phased in case 2/3. **b,** In the triploid genome, HP1 can be phased in case 4/5, HP2 can be phased in case 3/6, and HP3 can be phased in case 2/7. **c,** In the tetraploid genome, HP1 can be phased in case 8/9, HP2 can be phased in case 5/12, HP3 can be phased in case 3/14, and HP4 can be phased in case 2/15. Each color represented reads from the same haplotype in the merged files.

References

1 Grandi, F. C., Modi, H., Kampman, L. & Corces, M. R. Chromatin accessibility profiling by ATAC-seq. *Nat Protoc* **17**, 1518-1552, doi:10.1038/s41596-022-00692-9 (2022).
